# Exosomes derived from osteogenic tumor activate osteoclast differentiation and concurrently inhibit osteogenesis by transferring COL1A1‐targeting miRNA‐92a‐1‐5p

**DOI:** 10.1002/jev2.12056

**Published:** 2021-01-18

**Authors:** Lijuan Yu, Bingdong Sui, Weixiao Fan, Lin Lei, Lei Zhou, Liu Yang, Yanjun Diao, Yue Zhang, Zhuo Li, Jiayun Liu, Xiaoke Hao

**Affiliations:** ^1^ Institute of Laboratory Medicine Center of Chinese People's Liberation Army (PLA) Xijing Hospital Fourth Military Medical University (Air Force Medical University) Xi'an P.R. China; ^2^ Department of Clinical Laboratory Medicine Xijing Hospital Fourth Military Medical University (Air Force Medical University) Xi'an P.R. China; ^3^ Research and Development Center for Tissue Engineering School of Stomatology Fourth Military Medical University (Air Force Medical University) Xi'an P.R. China; ^4^ Department of Clinical Laboratory The First Affiliated Hospital of Xi'an Medical University Xi'an P.R. China; ^5^ College of Medicine Northwest University Xi'an P.R. China

**Keywords:** bone homeostasis, bone metastasis, *COL1A1*, exosomes, extracellular vesicles, miR‐92a‐1‐5p, prostate cancer

## Abstract

In patients with prostate cancer (PCa), bone lesions appear osteoblastic in radiographs; however, pathological fractures frequently occur in PCa patients, and bone resorption is observed in all metastatic lesions under histopathologic assessment. The mechanisms that balance the activities of osteoblasts and osteoclasts in PCa patients remain unclear. We unexpectedly discovered that PCa exosomes are critical mediators in the regulation of bone homeostasis that results in osteoclastic lesions and thereby promotes tumor growth in bone. We evaluated how exosomes derived from osteoblastic, osteoclastic, and mixed PCa cell lines affect osteoblast and osteoclast differentiation, revealing that all three types of PCa exosomes promoted osteoclastogenesis in vitro and induced osteolysis in vivo. Mechanistically, microRNAs (miRNAs) delivered by PCa exosomes were found to play several key roles in bone homeostasis. Among the delivered miRNAs, miR‐92a‐1‐5p, the most abundant miRNA, downregulated type I collagen expression by directly targeting *COL1A1*, and thus promoting osteoclast differentiation and inhibiting osteoblastogenesis. Furthermore, PCa exosomes also markedly reduced type I collagen expression in vivo. Our findings not only offer a novel perspective on tumor bone metastasis, where—contrary to our initial hypothesis—exosomes derived from an osteoblastic tumor induce osteoclast differentiation, but also suggest potential therapeutic targets for PCa bone metastasis.

## 1 INTRODUCTION

Prostate cancer (PCa) is the most common cause of new cancer cases and the second leading cause of cancer‐related deaths in males (Siegel et al., 2020). Bone is the most common target organ for high‐grade metastatic prostate cancer (Probert et al., 2019). Bone metastasis is detected at autopsy in ∼90% of all PCa patients and remains incurable; the 5‐year survival of patients is ∼30% (Cook et al., 2014; Probert et al., 2019; Siegel et al., 2020; Vessella & Corey, 2006). Tumor growth in bone is the result of tumor‐bone crosstalk that disrupts normal bone homeostasis between osteoblastic bone formation and osteoclastic bone resorption by tumor cells, thereby forming a pre‐metastatic niche. Previous research has shown that PCa cells crosstalk with the bone microenvironment through a variety of cytokines, chemokines, and some soluble proteins (Lin et al., 2018; Sottnik & Keller, 2013). PCa cells have been demonstrated to secrete both osteoblast‐stimulating factors (bone morphogenetic proteins, endothelin‐1, Wnt ligands) (Dai et al., 2005; Guise et al., 2003; Lee et al., 2011) and osteoclast‐stimulating factors (matrix metalloproteinases (MMPs), urokinase‐type plasminogen activator) (Sakamoto & Seiki, 2017; Shariat et al., 2007) and thus trigger bone formation, bone resorption, or both. These factors, however, neither entirely represent the tumor‐bone crosstalk nor completely account for the mechanisms underlying bone homeostasis in PCa bone metastasis.

Exosomes are extracellular vesicles that are 30–150 nm in size and originate from endosomes; they carry endosomal proteins such as Tsg101 or Alix (Boyiadzis & Whiteside, 2017; Cocucci & Meldolesi, 2015; Han et al., 2017). Intercellular and interorgan communication, mediated by exosomes, is a novel and powerful means of communication. PCa bone metastasis appears predominantly osteoblastic in radiographs, and the exosomal microRNAs (miRNAs) miR‐141‐3p and miR‐940 derived from PCa cells have been demonstrated to facilitate osteoblast differentiation and promote osteoblastic bone metastasis (Hashimoto et al., 2018; Ye et al., 2017). However, osteolytic lesions have also been detected in all patients with “osteoblastic” metastasis, which can explain the frequent pathologic fractures that occur in these patients (Roudier et al., 2008). Furthermore, the use of bisphosphonates and the RANKL inhibitor denosumab, potent inhibitors of bone resorption, represents the major treatment and prevention strategy for PCa bone metastasis, which underscores the notion that osteoclast differentiation cannot be ignored in the development of the disease. However, the precise roles of PCa exosomes and exosomal miRNAs in the regulation of bone homeostasis remain unclear.

To determine the functions of PCa exosomes in tumor‐bone crosstalk, we investigated the exosomes secreted by osteoblastic (MDA PCa 2b), osteoclastic (PC3), or mixed (C4‐2) human PCa cells. Specifically, we evaluated the roles of PCa exosomes in bone homeostasis, bone remodeling, and bone metastasis, and, notably, we identified a mechanism through which the exosomes produce their effect. This enhanced understanding of the bone‐tumor microenvironment could be key to the development of additional bone‐targeted therapies.

## 2 MATERIALS AND METHODS

### 2.1 Cell culture

In this study, we used four human PCa cell lines (MDA PCa 2b, C4‐2, PC3, and LNCap AI+F) and normal immortalized cells from the prostate (RWPE‐1 cells). MDA PCa 2b, C4‐2, PC3 and RWPE‐1 cells were obtained from American Type Culture Collection (ATCC, USA). LNCap AI+F cells (Xu et al., 2010) were a kind gift from Prof. Zhihua Tao at Zhejiang University, China. MDA PCa 2b cells were cultured in F12K medium (ATCC) containing 20% fetal bovine serum (FBS), according to ATCC instructions. C4‐2 and PC3 cells were cultured in RPMI 1640 medium (Gibco, USA) containing 10% FBS. RWPE‐1 cells were cultured in H‐DMEM (Gibco) containing 10% FBS. LNCap AI+F cells were cultured in F12K medium (ATCC) containing 10% charcoal stripped FBS (Gibco). The Raw264.7 cell line was maintained in H‐DMEM (Gibco) supplemented with 10% FBS. The MC3T3‐E1 clone 4 cell line was maintained in α‐MEM (Invitrogen, USA) containing 10% FBS. All culture media were replaced with fresh medium every 3 days and supplemented with 100 U/ml penicillin and 100 μg/ml streptomycin (HyClone, USA).

### 2.2 Exosome isolation, qualification and characterization

Exosomes were isolated from cell culture supernatants with 10% exosome‐depleted FBS (VivaCell Biosciences, China) through differential centrifugation, as described (Lötvall et al., 2014). After 48 h of culture, when cells were 90% confluent, 210 ml of the supernatant was harvested and centrifuged at 300 × *g* for 10 min, 2000 × *g* for 10 min, and then 10,000 × *g* for 30 min. Next, the supernatant was centrifuged at 120,000 × *g* for 70 min (Type 45 Ti Rotor, Beckman Coulter, USA), the pellet was resuspended in 35 ml of Phosphate buffered saline (PBS), and the sample was centrifuged again at 120,000 × *g* for 70 min. The supernatant was carefully removed, and the sample was resuspended in 200 μl of PBS. Exosomes isolated from human serum samples were obtained from six diagnosed PCa patients. Informed consent was obtained from all patients. Patient serum exosomes were isolated as described above and resuspended in 100 μl PBS. A BCA Protein assay kit (Pierce™, Thermo Fisher Scientific, USA) was used for exosome quantification, and exosome morphology was analysed using transmission electron microscopy (TEM) (Tecnai, USA), as previously described (Shelke et al., 2014). To examine exosome size distribution and particle concentration, nanoparticle tracking analysis (NTA) was performed using a ZetaView instrument (Particle Metrix, Germany).

### 2.3 Western blotting

Exosomes (10 μg) or proteins (30–50 μg) extracted from cells were diluted 1:4 (v/v) in a loading buffer (Beyotime, China), heated at 95°C for 10 min, loaded onto 10% gels (1.5 × 10 wells; Genscript, China), electrophoresed at 180 V for 50 min, and transferred to membranes by using the iBlot (Invitrogen) 8‐min program. For all subsequent antibody‐incubation and washing steps, a rocking platform was used. After blocking with a blocking buffer (Odyssey, USA) for 60 min, the membranes were incubated with primary antibodies at 4°C overnight, washed in TBST (3 × 5 min), incubated with secondary antibodies at room temperature for 60 min, washed with TBST, and then exposed to a detection reagent (Chemiluminescent HRP Substrate, Millipore, USA) for visualization. The primary antibodies were as follows: CD9 (Cell Signaling Technology, USA, 1:1000), Alix (Cell Signaling Technology, 1:1000), Annexin V (Cell Signaling Technology, 1:1000), HSP70 (Cell Signaling Technology, 1:1000), GM130 (Cell Signaling Technology, 1:1000), COL1A1 (Cell Signaling Technology, 1:1000), GAPDH (Cell Signaling Technology, 1:1000), β‐Actin (Cell Signaling Technology, 1:1000), and RANKL (Proteintech, China, 1:3000).

### 2.4 Uptake of exosomes

Exosomes (10 μg) were labelled using a PKH67 Green Fluorescent Cell Linker Kit (Sigma‐Aldrich), as per the manufacturer's protocol. Briefly, exosomes in PBS were added to 0.5 ml of Diluent C, and 4 μl of PKH67 was added to 0.5 ml of Diluent C; the two reaction mixtures were incubated in the dark at room temperature. After 4‐min incubation, 2 ml of 0.5% BSA was added to prevent excessive labelling, and then the labelled exosomes were resuspended in 35 ml of PBS and centrifuged 120,000 × *g* for 70 min to remove the residual dye. The recipient cells were mixed with the labelled exosomes and incubated in the dark. After aspirating the cell supernatant, the cells were washed with PBS once and fixed with 4% paraformaldehyde (PFA) for 10 min, and then the nucleus and the cytoskeleton were stained with DAPI and fluorophore‐conjugated phalloidin (to visualize F‐actin), respectively. Stained samples were examined using a confocal microscope (Nikon, Japan).

### 2.5 Conditioned medium (CM) preparation

CM was harvested as described (Liu et al., 2015). Briefly, 5 × 10^6^ MDA PCa 2b cells were cultured in 75‐cm^2^ cell‐culture flasks in F12K medium containing 20% FBS for 12 h, and then the medium was changed to 10 ml of F12K plus 0.5% FBS with/without 20 μM GW4869 (MCE, USA). After 48 h, the supernatant was collected and centrifuged at 2,500 rpm for 10 min to remove cell debris and then filtered (0.2‐μm filter, Millipore) before use in experiments.

### 2.6 In vitro osteoclast induction of Raw264.7 cells

Osteoclast induction of Raw264.7 cells was performed as described (Sun et al., 2016), with modifications. Briefly, Raw264.7 cells were seeded into 96‐well plates at a density of 1 × 10^4^ cells/well, allowed to attach for 24 h, and then cultured in the presence or absence of recombinant murine sRANKL (100 ng/ml, PeproTech, USA) in combination with exosomes or miRNAs for 6 days. The medium was changed every 2 days, and after 6 days of culture, osteoclast differentiation was assessed by means of Trap staining and Trap‐activity detection (Beyotime).

### 2.7 In vitro osteoclast induction of bone marrow macrophages (BMMs)

We prepared bone marrow cells and cultured BMMs according to previously reported protocols (Li et al., 2016), with minor modifications. Briefly, bone marrow cells were isolated from the tibia and femur of 8–12‐week‐old male mice and cultured in α‐MEM (Invitrogen) containing 10% FBS for 24 h to generate BMMs. To produce osteoclasts, BMMs were cultured in α‐MEM supplemented with recombinant murine M‐CSF (25 ng/ml, PeproTech) and recombinant murine sRANKL (50 ng/ml, PeproTech) with or without treatment with exosomes or miRNAs for 6 days. The culture medium was changed every 2 days until Day 6, and then Trap staining was performed.

### 2.8 Trap staining

Trap staining for osteoclasts was performed according to the manufacturer's instructions (Sigma‐Aldrich), with minor modifications. Cells were fixed with Fixative Solution (a mixture of 4.05 ml of acetone, 1.55 ml of citrate solution, and 0.5 ml of formaldehyde) for 3 min at room temperature and then washed with deionized water once. Next, 0.05 ml of Fast Garnet GBC base solution and 0.05 ml of sodium nitrite solution were mixed for 2 min and then mixed with another solution containing 4.5 ml of 37°C deionized water, 0.05 ml of naphthol AS‐BI phosphate solution, 0.2 ml of acetate solution, and 0.1 ml of tartrate solution. Lastly, the fixed cells were incubated in the final mixed solution for 1 h at 37°C.

### 2.9 In vitro osteogenic induction of MC3T3‐E1 cells

MC3T3‐E1 cells were seeded at 1 × 10^4^ cells/well into 24‐well plates on Day 0, cultured in α‐MEM until 80%–90% confluence, and then changed into an osteogenic differentiation medium (Cyagen Biosciences, China). After induction for 21 days, Alizarin Red S staining was performed as previously reported (Gao et al., 2019). Briefly, on Day 21, cells were fixed with 4% PFA for 15 min, incubated with Alizarin Red S (Cyagen Biosciences) for 3 min, and washed with deionized water. To quantify mineralization, 2% hexadecyl pyridinium chloride monohydrate solution was added and the 490‐nm absorbance was measured. For alkaline phosphatase (ALP) staining, cells were stained using a BCIP/NBT Alkaline Phosphatase Color Development Kit (Beyotime) after 7 days of osteogenic induction, and ALP activity was measured using an Alkaline Phosphatase Assay Kit (Beyotime), according to the manufacturer's instructions.

### 2.10 Library preparation and sequencing

Small‐RNA sequencing was conducted by a commercial service (RiboBio, China). Briefly, total RNAs were extracted from MDA PCa 2b exosomes and RWPE‐1 exosomes, and the quality and concentration of the RNAs were checked using an Agilent 2200. After reverse‐transcription‐PCR amplification, small‐RNA libraries were prepared and sequenced, with raw reads being collected using an Illumina HiSeq 2500 platform.

### 2.11 Differential expression analysis of miRNAs

To validate miRNAs identified through RNA sequencing, RNA was extracted using a Total Exosome RNA and Protein Isolation Kit (Invitrogen) and then a Mir‐X™ miRNA First Strand Synthesis kit (Takara, Japan) and SYBR® Premix Ex Taq™ II (Takara) were used for qPCR‐based measurement of the expression levels of miR‐92a‐1‐5p, miR‐148a‐3p, and miR‐375 in exosomes. U6 and cel‐miR‐39 were used as controls. The miRNA qPCR primer sets were purchased from RiboBio and mRNA qPCR primers were synthesized by Sangon (Shanghai, China). Table S1 lists the qPCR primers used in this study.

### 2.12 Target‐gene prediction and KEGG/GO pathway enrichment

Targetscan, miRanda, miRWalk, and miRDB were used for miRNA target‐gene prediction. We used the tool DIANA to discover the pathways enriched by the miRNA targets (http://www.microrna.gr/miRPathv3) (Vlachos et al., 2015), and then used David 6.8 Bioinformatics Resources (https://david.ncifcrf.gov/) to analyse the functional annotations (Huang et al., 2009).

### 2.13 Lentivirus and oligonucleotide transfection

Lentiviral plasmids encoding miR‐92a‐1‐5p and a negative control were constructed by HANBIO (Shanghai, China). Raw264.7 and MC3T3‐E1 cells were transfected with the lentivirus (pHB‐U6‐mir‐92a‐1‐5p‐EF1‐LUC‐PURO) at a multiplicity of infection of 10, after which selection was performed using 4 μg/ml puromycin for 6 days. Synthetic miRNA mimics, miRNA inhibitors, and control miRNAs (RiboBio) were synthesized and the oligonucleotides were transfected using a RiboFECT CP Transfection Kit (RiboBio).

### 2.14 Luciferase reporter assay

Cells were co‐transfected with luciferase vectors containing wild‐type or mutant 3ʹ‐UTR of *COL1A1* and miR‐92a‐1‐5p mimics or miR‐Control by using Lipofectamine 3000 (Invitrogen). Luciferase activity was measured using a Dual‐Luciferase Reporter Assay System (Beyotime) at 48 h after transfection.

### 2.15 COL1A1 knockdown by siRNA

The siRNA oligonucleotides for mouse COL1A1 and siRNA control were purchased from Ribobio. MC3T3‐E1 and Raw264.7 cells were transfected using a RiboFECT CP Transfection Kit (RiboBio), followed by validation of mRNA change at 36 h after transfection using qPCR and protein change at 48 h using western blotting.

### 2.16 Animal studies

All experiments were performed following the animal welfare guidelines approved by the Intramural Animal Use and Care Committee at Air Force Medical University. All efforts were made to reduce animal suffering to the extent possible. Male BALB/C nude mice (6 weeks old; 18–20 g) were purchased from SJA Laboratory Animal Co. (Changsha, China) and randomly assigned to different treatment groups. The mice were acclimatized to the specific‐pathogen‐free animal housing facility for one week before the experiments were started.

### 2.17 Exosomes labelling and tracking in vivo

To visualize the target organ and the metabolism process of exosomes in vivo, exosomes were labelled according to the instructions of the manufacturer, Life Technologies (USA), with modifications. Briefly, 100 μg of exosomes were incubated with Vybrant DID (1:1000 in PBS) in the dark for 20 min, and then the labelled exosomes were washed in 35 ml of PBS, with centrifugation at 120,000 × *g* for 90 min, to remove the excess dye. Next, the Vybrant DID‐labelled exosomes were injected into the tail vein of BALB/C nude mice (7 weeks old, *n* = 3 per group/time point, dosage per mouse: 100 μg of exosomes, in 150 μl of PBS), and PBS with/without Vybrant DID was used as the control in all experiments. At 4, 24, and 48 h after injection, the mice and the harvested tissues were subject to in vivo and ex vivo imaging. Fluorescence intensity was determined using an IVIS Spectrum system and Living Image Software (PerkinElmer, USA).

### 2.18 Enrichment of bone marrow cells and flow cytometry

To verify that the detected fluorescence was from bone marrow cells that had internalized Vybrant DID‐labelled exosomes, mice were euthanized at 24 h post‐injection and the femur and tibia were flushed with PBS by using a syringe attached with a 27‐G needle. Cell suspensions were passed through a 70‐μm cell strainer (BD Biosciences, USA) and red blood cells were lysed using a Red Blood Cell Lysis Buffer (Beyotime), and then bone marrow cells were assessed using flow cytometry.

### 2.19 Immunofluorescence staining of tibia

To visualize the colocalization of Vybrant DID‐labelled exosomes and bone stromal cells, immunofluorescence analysis of the tibia was performed as reported (Sui et al., 2016), with modifications. The left tibia was isolated at sacrifice 24 h after injection of labelled exosomes, fixed in 4% PFA, decalcified rapidly, and embedded in paraffin wax; subsequently, specimens were sectioned at 3‐μm thickness by using a tissue slicer (Leica, Germany) away from light. Without quenching the spontaneous fluorescence (red) from Vybrant DID‐labelled exosomes, nuclei and bone stromal cells in the bone were stained with DAPI (blue) and anti‐osteocalcin (OCN)/RANKL/cathepsin K (CTSK)/TRAP (green), respectively. Immunofluorescence labelling for COL1A1 and type I collagen was performed using a routine staining method. Samples were examined using a fluorescence microscope (Nikon) or scanned using a case viewer system (3DHISTEC, Hungary).

### 2.20 Micro‐computed tomography (micro‐CT) analysis

To evaluate bone mass, a micro‐CT system (GE Healthcare, USA) was used as reported (Bouxsein et al., 2010; Sui et al., 2016), with modifications. Mice were sacrificed and the right tibia was removed and fixed in 4% PFA for 24 h, and then 7‐mm blocks of the specimen were prepared. Subsequently, the specimens were imaged using a 120‐min micro‐CT scan, at a resolution of 8 μm, a current of 80 μA and a voltage of 80 kV. Micview V2.1.2 software was used for data analysis, and the following parameters were quantified: bone mineral density (BMD), bone volume per tissue volume (BV/TV), bone surface per bone volume (BS/BV), trabecular number (Tb.N), trabecular thickness (Tb.Th), and trabecular separation (Tb.Sp) (Bouxsein et al., 2010).

### 2.21 Mouse bone marrow education

Mice (7 weeks old, *n* = 3–6 per group) were injected with a small dose of exosomes, as previously reported (Costa‐Silva et al., 2015; Peinado et al., 2012; Wen et al., 2016); 10 μg of total exosomes (in 100 μl of PBS) were injected into the tail vein thrice weekly for 4 weeks, and after this initial treatment, 50 μg of exosomes (in 150 μl of PBS) were injected once a week. PBS was used as the control. On Days 28, 42, and 56, mice were sacrificed and specimens were collected for different experiments.

### 2.22 Intratibial (i.t.) injection

We performed i.t. injections as reported (Berlin et al., 1993; Wu et al., 1998), with minor modifications. Briefly, after the 4‐week bone marrow education, BALB/C nude mice (*n* = 6 per group) were anesthetized using sodium pentobarbital and the right legs were cleaned with iodine solution. PCa cells infected with pHB‐U6‐mir‐92a‐1‐5p‐EF1‐LUC‐PURO lentivirus (HANBIO) were aspirated into a 25‐μl Hamilton syringe (Alltech Associates, Switzerland) mounted with a 27‐G needle, and the needle was inserted into the cortex of the anterior tuberosity with a rotating “drill‐like” movement and inserted down 3–5 mm once the bone cortex was traversed (Berlin et al., 1993). Subsequently, 25 μl of the cell suspension (2.5 × 10^6^ cells/inoculum) was slowly injected into the bone marrow space, after which the needle was removed from the bone, and bone wax was applied to the injection area.

### 2.23 Orthotopic injection

Male BALB/C nude mice (7 weeks old, *n* = 6 per group) were anesthetized using sodium pentobarbital, the abdomen was surgically prepared with iodine solution, and a surgical incision was made in the lower abdomen to expose the prostate. Subsequently, miR‐92a‐1‐5p‐overexpressing MDA‐PCa‐2b‐luc cells (10^6^ cells in 10 μl) were injected into each dorsal prostate lobe by using a Hamilton syringe attached with a 27‐G needle.

### 2.24 In vivo imaging

Tumor growth was detected using an IVIS Spectrum system (Perkin Elmer), as previously reported (Dai et al., 2019). Briefly, D‐luciferin at a working dose of 150 mg/kg (in 100 μl of PBS) was intraperitoneally injected, and 1.5% isoflurane was used for anesthetization before imaging. Tumor growth and tumor burden were measured based on bioluminescence imaging by using Living Image Software (Perkin Elmer).

### 2.25 Statistical analysis

All data are presented as means ± standard deviation. Comparisons were performed using *t* test and one‐way ANOVA as appropriate, by using GraphPad Prism version 7.0. *P* < 0.05 was considered statistically significant (*, *P* < 0.05; **, *P* < 0.01; ***, *P* < 0.001).

## 3 RESULTS

### 3.1 Characteristics of exosomes derived from osteoblastic, osteoclastic, and mixed PCa cells

**FIGURE 1:**
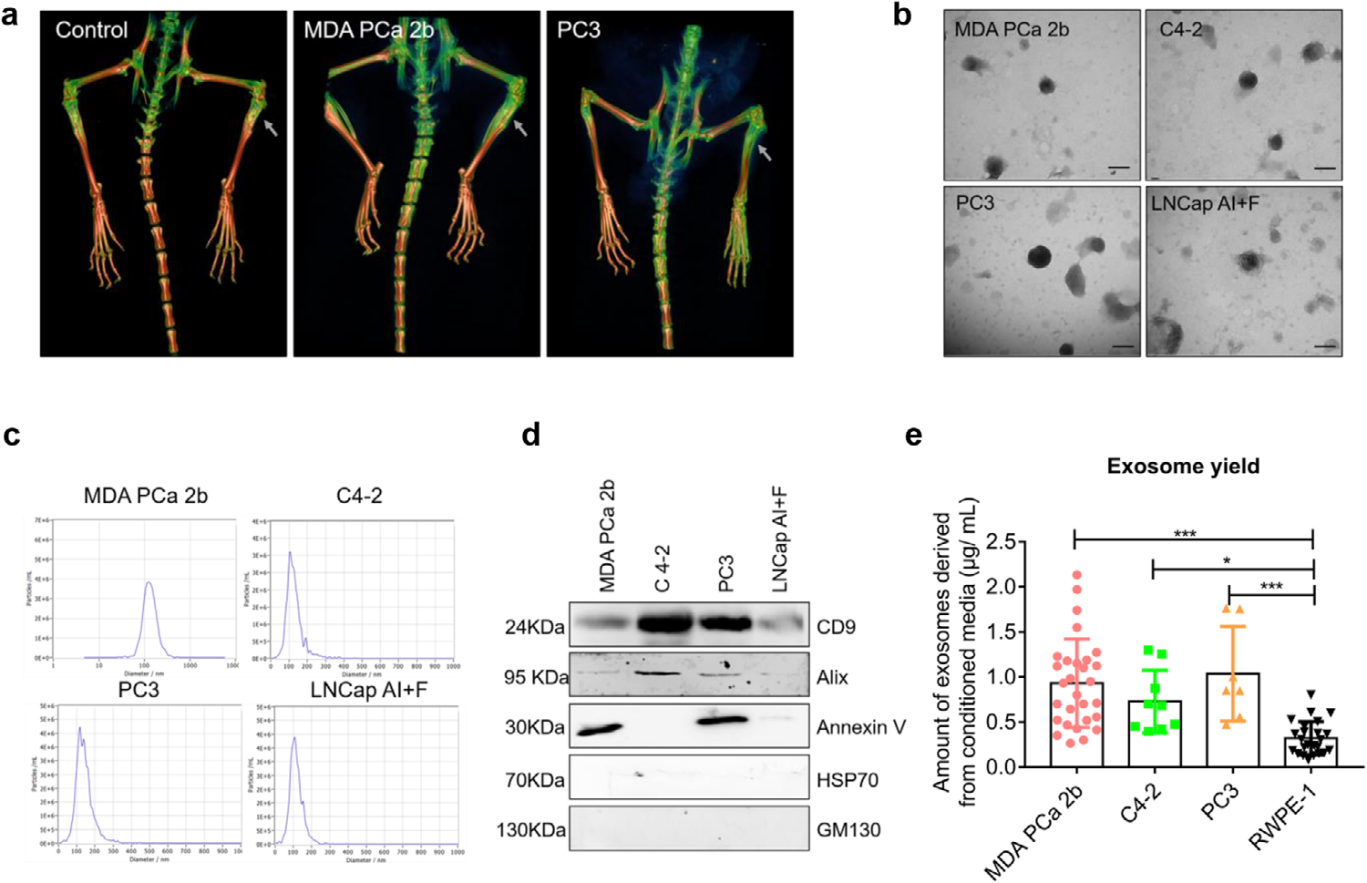
Characteristics of PCa exosomes. (a) Representative 3D‐reconstruction images from micro‐CT analysis of bone at 8 weeks after i.t. injection of 1 × 10^7^ tumor cells into BALB/C nude mice. Osteoblastic and osteoclastic lesions are shown to be induced by MDA PCa 2b cells and PC3 cells, respectively. (b) Representative TEM images of PCa exosomes. Homogenous PCa exosomes with an ovoid morphology are shown. Scale bar = 100 nm. (c) Average size distribution of PCa exosomes in the range 90–130 nm by NTA. (d) Indicated proteins CD9, Alix, Annexin V, HSP70, and GM130 were assessed by western blotting from 10 μg of exosomes. (e) Comparison of exosome yield by BCA protein assay. Amounts of exosomes are shown markedly higher in all PCa groups than normal prostate cells (RWPE‐1) group. Data were analysed using one‐way ANOVA with multiple comparisons test. *P < 0.05; ** *P* < 0.01; ***P < 0.001

PCa cell lines are frequently divided into three groups: osteoblastic, osteolytic, and mixed cell lines. According to previous studies, MDA PCa 2b cells exhibit the osteoblastic phenotype of PCa cells and induce osteogenesis when implanted into the mouse tibia (Figure 1a) (Navone et al., 1997). By contrast, two other PCa cell lines, PC3 and DU145, are known to induce osteolytic lesions in bone metastasis (Figure 1a) (Fisher et al., 2002; Russell & Kingsley, 2003). PCa cell lines featuring a mixed phenotype, LNCap and its derivative C4‐2/C4‐2B, produce mixed lesions (Thalmann et al., 1994).

We isolated exosomes derived from osteoblastic, osteoclastic, and mixed PCa cells and then characterized single vesicles within each mixture by using TEM. The exosomes isolated from MDA PCa 2b, C4‐2, PC3, and LNCap AI+F cells appeared homogenous and displayed an ovoid morphology (Figure 1b). Moreover, examination of the size distribution of exosomes by using NTA revealed that the majority of the population was in the ∼90–130‐nm range (Figure 1c), which is within the characteristic diameter range of 30–150 nm of these vesicles. As recommended (Lötvall et al., 2014), the values acquired using NTA were compared with the TEM results to verify that the isolated vesicles were not non‐membranous particles of a similar size. To further confirm that exosomes had been isolated, we used western blotting to test for the presence of several “exosome markers”: a transmembrane protein (CD9), three cytosolic proteins (Annexin V, Alix, HSP70), and an intracellular protein (GM130) (Figure 1d). All exosomes isolated from the four types of PCa cells expressed CD9 and Alix, although the exosomes showed clear differences in the enrichment of the proteins; furthermore, the exosomes from MDA PCa 2b, PC3, and LNCap AI+F cells were positive for Annexin V, whereas C4‐2 exosomes were negative for this marker. Our results also agree with the report that GM130 is not expected to be enriched in exosomes (Lötvall et al., 2014). HSP70 was not expressed in PCa exosomes, which is consistent with our previous results (data not shown). Collectively, these results on the morphology, size, and marker expression of the isolated vesicles confirmed that the vesicles were exosomes. Lastly, quantification of the isolated exosomes by using the BCA method revealed that the exosome yield differed markedly between PCa cells and normal prostate cells (RWPE‐1), with PCa cells producing ∼3‐fold more exosomes (Figure 1e).

### 3.2 PCa exosomes target bone and are internalized by bone stromal cells in vivo

**FIGURE 2:**
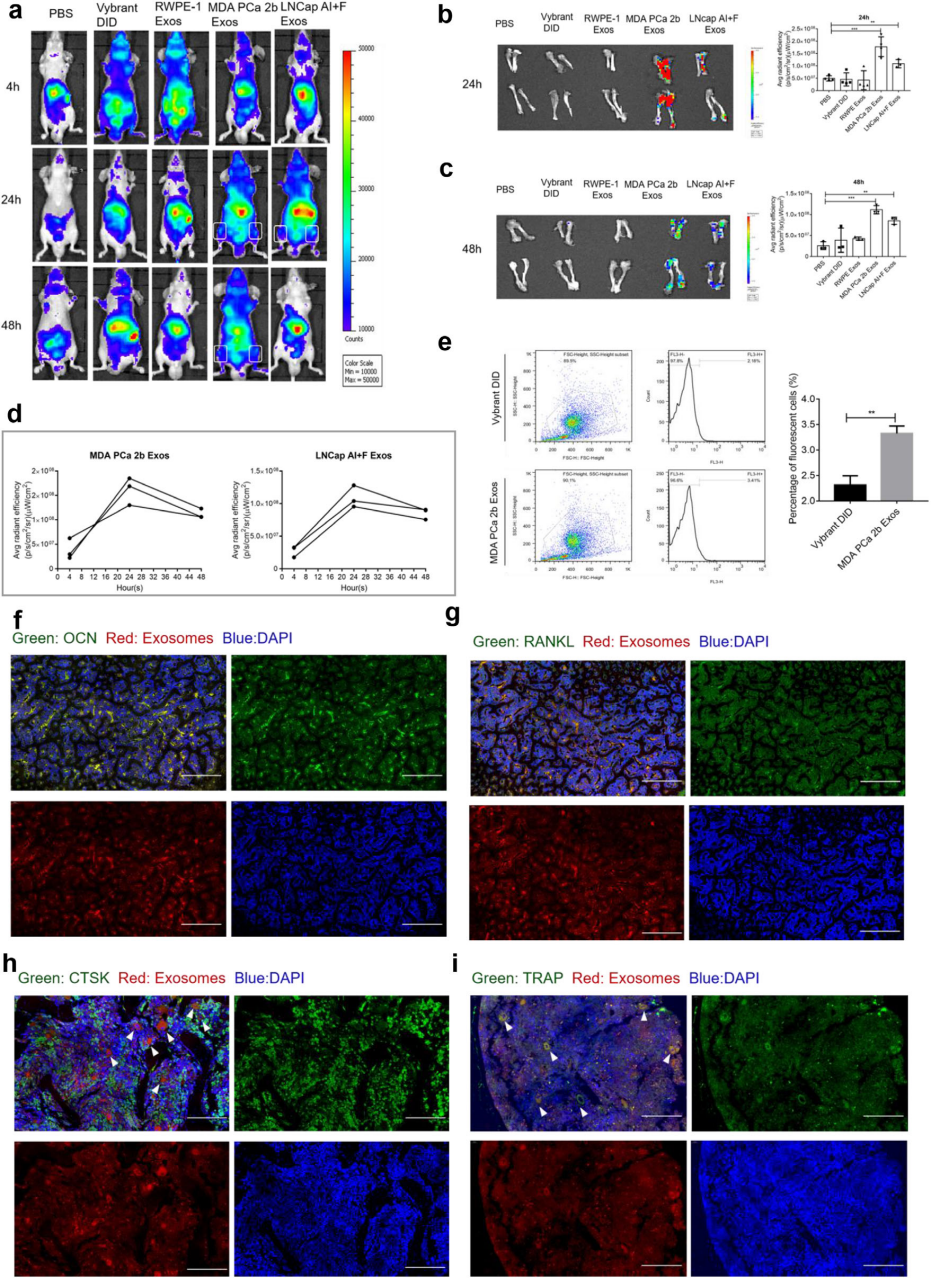
PCa exosomes target bone in vivo. BALB/C nude mice received a single injection through the tail vein of Vybrant DID‐labelled exosomes (100 μg in 150 μl of PBS), PBS, or Vybrant DID. (a) Representative in vivo images at 4, 24, and 48 h. Exosomes are shown to target on bone in vivo at 24 and 48 h only in PCa Exos groups. (b–c) Ex vivo images and fluorescent intensity comparison at 24 and 48 h after injection. Fluorescence intensity of PCa Exos groups was significantly increased than PBS group at each time point. (d) Dynamics of bone fluorescence intensity at 4, 24, and 48 h with fluorescence intensity peaking at 24 h. (e) Comparison of fluorescence intensity from flushed bone marrow cells by flow cytometry analysis, indicating the fluorescence of PCa‐Exos groups was from the uptake of Vybrant DID‐labelled exosomes. (f–g) Colocalization of Vybrant DID‐labelled exosomes with OCN and RANKL at 24 h after tail‐vein injection, showing PCa exosomes were internalized by osteoblasts in vivo. Scale bars = 100 μm. (h‐i) Colocalization of Vybrant DID‐labelled exosomes with CTST and TRAP at 24 h after tail‐vein injection, showing PCa exosomes were internalized by osteoclasts in vivo. Scale bars = 50 μm. Data were analysed using *t* test (e) and one‐way ANOVA with multiple comparisons test (b‐c). *P < 0.05; **P < 0.01; ***P < 0.001

To elucidate the roles of PCa exosomes, it is critical to first determine the distribution and metabolism of PCa exosomes in vivo. We determined the tissue distribution of PCa exosomes by labelling the exosomes with Vybrant DID, a lipid‐based fluorescent dye, injecting the labelled exosomes into the tail vein of BALB/C nude mice (7 weeks old, *n* = 3 per group/time point), and assessing the biodistribution of the exosomes in vivo and ex vivo at 4, 24, and 48 h post‐injection. The in vivo fluorescence signal was only observed in the bones of the PCa‐Exos groups (MDA PCa 2b Exos group and LNCap AI+F Exos group), but not the RWPE‐1 Exos group which did not localize to the bone, indicating this bone target is PCa specific (Figure 2a). Next, bones were harvested and their fluorescence intensity was measured ex vivo; at both 24 and 48 h, the average fluorescence intensity of the PCa‐Exos groups was elevated substantially (Figure 2b,c). Examination of the dynamics of bone fluorescence intensity in the PCa‐Exos groups (Figure 2d) revealed that the intensity peaked at 24 h and then decreased drastically by 48 h. Therefore, we selected the 24‐h time point for further analysis; this time point is the same as that used in most studies (Inder et al., 2014; Wen et al., 2016), although (to our knowledge) the peaking of the signal at this time point has not been previously verified. We also evaluated the fluorescence signal from various soft organs (heart, liver, spleen, kidney, stomach, and intestine) and found no differences between the groups (Figure S1a). At 24 h post‐injection, no pathological changes were detected in these soft organs in the PCa‐Exos groups, which suggested that the exosomes are appropriately targeted and show almost no systemic toxicity (Figure S1b). Overall, these results suggested that PCa‐derived exosomes, relative to normal exosomes, exhibit a high tendency for bone‐specific targeting.

Next, we determined whether the observed fluorescence was derived specifically from cells that had internalized the fluorescently labelled exosomes. Flow cytometry was used to measure the fluorescence signal of bone marrow cells flushed from long bones; the fluorescence level was higher in the MDA PCa 2b‐Exos group than in the Vybrant DID group (Figure 2e), which indicated that the red fluorescence was from bone marrow cells that had internalized PCa exosomes. We also sought to identity the types of bone marrow cells that had taken up the exosomes. Without quenching the spontaneous fluorescence from the labelled MDA PCa 2b exosomes (red), bones were subject to immunofluorescence labelling by using anti‐OCN/RANKL/CTSK/TRAP (green), and nuclei were stained with DAPI (blue). The fluorescence of the labelled exosomes colocalized with the signal from osteoblasts and osteoclasts (white arrows indicated), which confirmed that PCa exosomes were internalized by bone stromal cells following tail‐vein injection (Figure 2f‐i).

### 3.3 PCa exosomes are internalized rapidly by Raw264.7 cells and facilitate osteoclast differentiation in vitro

**FIGURE 3:**
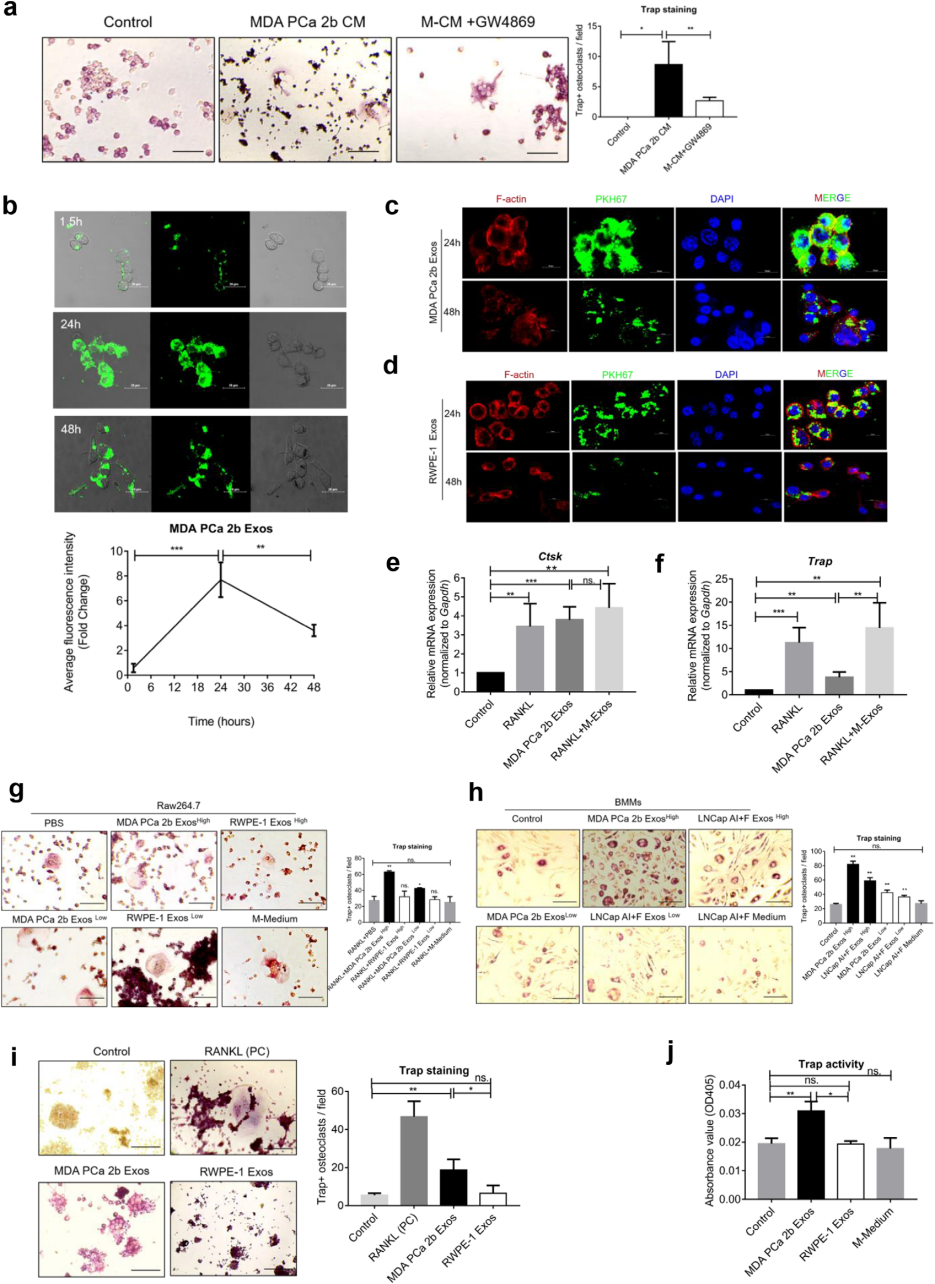
PCa exosomes facilitate osteoclast differentiation in vitro. (a) Representative Trap‐staining images (left, scale bar = 100 μm) and quantification (right) of Trap^+^ osteoclasts. At 2 days after culture with RANKL (100 ng/ml), Raw264.7 cells were cultured without treatment (Control) or cultured with 10% CM from either MDA PCa 2b cells (MDA PCa 2b CM) or GW4869‐treated MDA PCa 2b cells (M‐CM+GW4869). (b) PKH67‐labelled MDA PCa 2b exosomes were cultured with Raw264.7 cells at 37°C for the indicated times. Representative fluorescence microscopy images (upper) and total cellular fluorescence (lower) at each time point quantified using ImageJ. Scale bar = 20 μm. (c–d) Raw264.7 cells were incubated with PKH67‐labelled exosomes for 24 or 48 h, and then the cells were stained to visualize nucleus (with DAPI) and F‐actin. Osteoclast differentiation was indicated by cell fusion induced by MDA PCa 2b Exos. Scale bar = 10 μm. (e–f) qPCR analysis of Ctsk and Trap mRNAs in Raw264.7 cells after culture with 100 ng/ml RANKL and (or) 20 μg/ml MDA PCa Exos for 6 days. The medium was changed every other day. (g) Trap staining of Raw264.7 cells after treatment with high or low concentration of exosomes (50 or 25 μg/ml, respectively) in the presence of 100 ng/ml RANKL. Scale bar = 100 μm. *, compared with PBS group. (h) Trap staining of BMMs after treatment with 50 or 25 μg/ml exosomes in the presence of 50 ng/ml RANKL and 30 ng/ml M‐CSF. Scale bar = 100 μm. *, compared with Control group. (i) Trap staining of Raw264.7 cells after incubation with exosomes alone for 6 days. RANKL: positive control. Scale bar = 100 μm. (j) Trap activity measured in osteoclasts after treatment with 40 μg/ml exosomes. Data were analysed with one‐way ANOVA with multiple comparison test. *P < 0.05; **P < 0.01; ***P < 0.001

Based on the PCa‐exosome organotropism detected here with bone and bone stromal cells, we investigated whether PCa exosomes are involved in osteoclast differentiation. First of all, we prepared MDA PCa 2b CM from cells cultured with or without the exosome inhibitor GW4869. The decrease of exosome yield by GW4869 was confirmed using BCA Protein Assay and NTA (Figure S2a and S2b). After priming with 100 ng/ml RANKL for 2 days, osteoclast differentiation of Raw264.7 cells was increased following culture with MDA PCa 2b CM for 48 h, but this effect was reversed by GW4869 (Figure 3a). We further observed that MDA PCa 2b exosomes were rapidly internalized by Raw264.7 cells (Figure 3b, upper); the uptake occurred within only 90 min of culture, which was a shorter time than that reported for other recipient cells (Kalra et al., 2019; Raimondi et al., 2015). The results of ImageJ analysis showed that the uptake peaked at 24 h and then decreased markedly by 48 h (Figure 3b, lower). Next, we sought to examine whether cell fusion occurred during osteoclast differentiation induced by PCa exosomes. After culture for 24 or 48 h with exosomes alone, Raw264.7 cells were fixed with 4% PFA and stained for F‐actin and nuclei (with DAPI) and examined under a confocal microscope; our result showed that MDA PCa 2b exosomes induced the fusion of Raw264.7 cells at both 24 and 48 h (Figure 3c), whereas exosomes derived from normal RWPE‐1 cells did not induce cell fusion (Figure 3d).

RANKL has been reported to play a crucial role in osteoclast differentiation (Boyle et al., 2003). BMMs (bone marrow macrophages) and Raw264.7 cells (murine macrophage cell line) are pre‐osteoclasts, often used for osteoclastogenesis. Here, we first checked for Trap^+^ osteoclasts after culturing with various concentrations of RANKL (0, 20, 50, 100, 150, 200 ng/ml) and then selected 100 ng/ml RANKL for further studies (Figure S2c). To observe the mRNA expression of osteoclast markers, Raw264.7 cells were incubated with 100 ng/ml RANKL and (or) 20 μg/ml MDA PCa exosomes for 6 days. Next, qPCR analysis revealed that MDA PCa 2b exosomes markedly induced the mRNA expression of Cstk and Trap in Raw264.7 cells after a 6‐days incubation (Figure 3e,f). The synergistic effect by RANKL and exosomes was observed by Trap mRNA but not observed significantly by Ctsk mRNA, indicating that PCa exosomes may promote osteoclastogenesis in a RANKL independent manner (Figure 3e,f). Furthermore, our results demonstrated that MDA PCa 2b exosomes induce osteoclast differentiation of Raw264.7 cells in the presence of RANKL in a dose‐dependent manner (Figure 3g). We also verified that MDA PCa 2b exosomes induced the osteoclast differentiation of BMMs in the presence of RANKL and M‐CSF (Figure 3h). We further confirmed that exosomes from mixed C4‐2/LNCap AI+F cells and osteoclastic PC3 cells also induced the osteoclast differentiation of BMMs (Figure S2d, Figure 3h) and Raw264.7 cells (Figure S2e). To determine whether PCa exosomes alone can induce osteoclast differentiation, we cultured Raw264.7 cells with exosomes for 6 days and then performed Trap staining and quantified Trap^+^ osteoclasts (Figure 3i). Unexpectedly, osteoclastogenesis was also induced by PCa exosomes in the absence of RANKL (Figure 3j). Next, our results inspired us to determine whether RANKL is transferred by PCa exosomes. As examined by western blotting, RANKL was expressed at low level in all three types of PCa cells and no expression was found in PCa exosomes (Figure S2f). qPCR analysis also demonstrated that RANKL was lowly expressed in PCa cells with an ∼28 ct value in 0.2 μg total RNA (Figure S2g). This result contradicts the accepted notion that RANKL is necessary for osteoclast differentiation, but is in accord with the results shown in Figure 3c. In summary, we used Raw264.7 cells and BMMs, two commonly used models for osteoclast differentiation (Song et al., 2019), to verify that exosomes derived from osteoblastic, osteoclastic, and mixed PCa cells can induce osteoclast differentiation both in the absence and presence of RANKL.

### 3.4 PCa exosomes inhibit osteoblastogenesis of MC3T3‐E1 cells in vitro

To further analyse the observed exosome internalization by bone stromal cells, we investigated the potential effects of the exosomes on osteoblasts. We examined the differentiation of MC3T3‐E1 cells, which represent a murine pre‐osteoblast cell line and can differentiate into mature osteoblasts in osteogenic induction medium containing β‐glycerophosphate and ascorbic acid (Wang et al., 1999).

**FIGURE 4:**
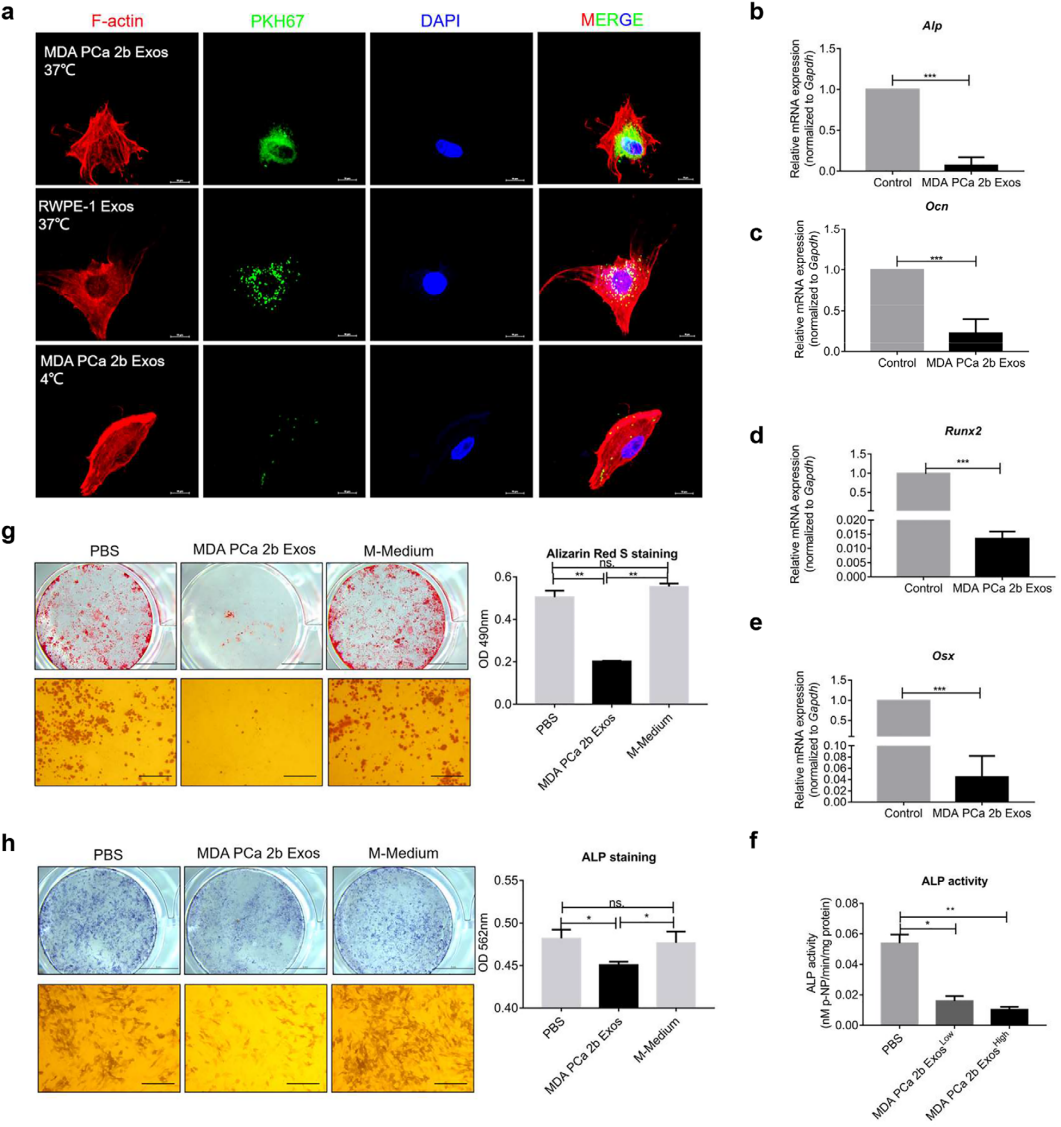
PCa exosomes inhibit osteoblast differentiation in vitro. (a) Confocal microscopy images of MC3T3‐E1 cells treated for 4 h with 20 μg/ml PKH67‐labelled exosomes at 37°C and 4°C. Exosomes internalization by MC3T3‐E1 at 37°C after 4 h is shown; the internalization was inhibited at 4°C. Scale bar = 40 μm. (b–e) qPCR analysis of osteoblast markers after a 6‐day treatment with 20 μg/ml MDA PCa 2b exosomes. The normal medium was changed every 3 days. Decreases in all the indicated osteoblast markers after the treatment are shown. (f) ALP activity decreased after a 7‐day treatment with 20 μg/ml MDA PCa 2b exosomes (low concentration) or 40 μg/ml MDA PCa 2b exosomes (high concentration). (g) Alizarin Red S staining after a 21‐day treatment with 20 μg/ml MDA PCa 2b exosomes, PBS, or the same volume of MDA PCa 2b medium in osteogenic induction medium. Reduced capacity for mineralized nodule formation is shown in MDA PCa 2b Exos group. Scale bars = 5 μm (upper) and 100 μm (lower). (h) ALP staining after a 7‐day treatment with 20 μg/ml MDA PCa 2b exosomes, PBS, or the same volume of MDA PCa 2b Medium in osteogenic induction medium. Decrease is shown in MDA PCa 2b Exos group. Scale bars = 5 μm (upper) and 100 μm (lower). Data were analysed using *t* test (b‐e) and one‐way ANOVA with multiple comparisons test (f‐h). *P < 0.05; **P < 0.01; ***P < 0.001

PCa cell‐derived exosomes labelled with PKH67 were internalized by MC3T3‐E1 cells after 4‐h incubation at 37°C (Figure 4a); the internalization was inhibited when cells were incubated at 4°C, which indicated that exosome uptake by the recipient cells was a biologically active process. Next, we cultured the cells with the exosomes in normal medium for 6 days and then measured the mRNA expression of the osteoblastic markers Alp, Ocn, Runx2, and Osx (Figure 4b–e); the results confirmed that all four markers were drastically downregulated, which indicated that the osteoblastogenesis of MC3T3‐E1 cells was inhibited by MDA PCa 2b exosomes. Moreover, in agreement with the qPCR data, the MDA PCa 2b exosomes decreased ALP activity at high or low concentration (Figure 4f). As a classical and recommended experiment of osteogenic differentiation, Alizarin Red S staining was also performed: When MC3T3‐E1 cells were cultured with MDA PCa 2b exosomes in osteogenic induction medium for 21 days, the cells showed markedly reduced capacity for mineralized nodule formation (Figure 4g). Furthermore, in agreement with the ALP mRNA expression, ALP staining was decreased after the cells were cultured with MDA PCa 2b exosomes (Figure 4h). We also examined the ALP activity and ALP staining of MC3T3‐E1 cells after treatment with exosomes derived from other PCa cells (C4‐2, PC3, and LNCap AI+F) (Figure S3). Overall, our results presented in the preceding subsection demonstrated that PCa exosomes accelerated osteoclast differentiation, and our results in this subsection further showed that PCa exosomes inhibited osteoblastogenesis.

To further strengthen our results on the role of PCa cell‐derived exosomes on bone homeostasis, we assessed the effect of exosomes isolated from the serum of six PCa patients (three localized PCa and three PCa with bone metastasis) on the osteoclast differentiation of BMMs in three independent experiments. The exosome yield was assessed by BCA protein assay; the amounts of exosomes from serum of PCa with bone metastasis were significantly higher (Figure S6a). Exosomes from localized PCa and PCa with bone metastasis both promoted osteoclast differentiation of BMMs (Figure S6b). Moreover, we discovered that exosomes from PCa with bone metastasis had a stronger ability to promote osteoclast differentiation in all three independent experiments. We also investigated the potential of bone metastatic PCa patient‐derived exosomes to inhibit osteoblastogenesis; ALP staining assay revealed a trend toward reduction (Figure S6c).

### 3.5 Osteogenic tumor‐derived exosomes induce osteolysis in vivo

**FIGURE 5:**
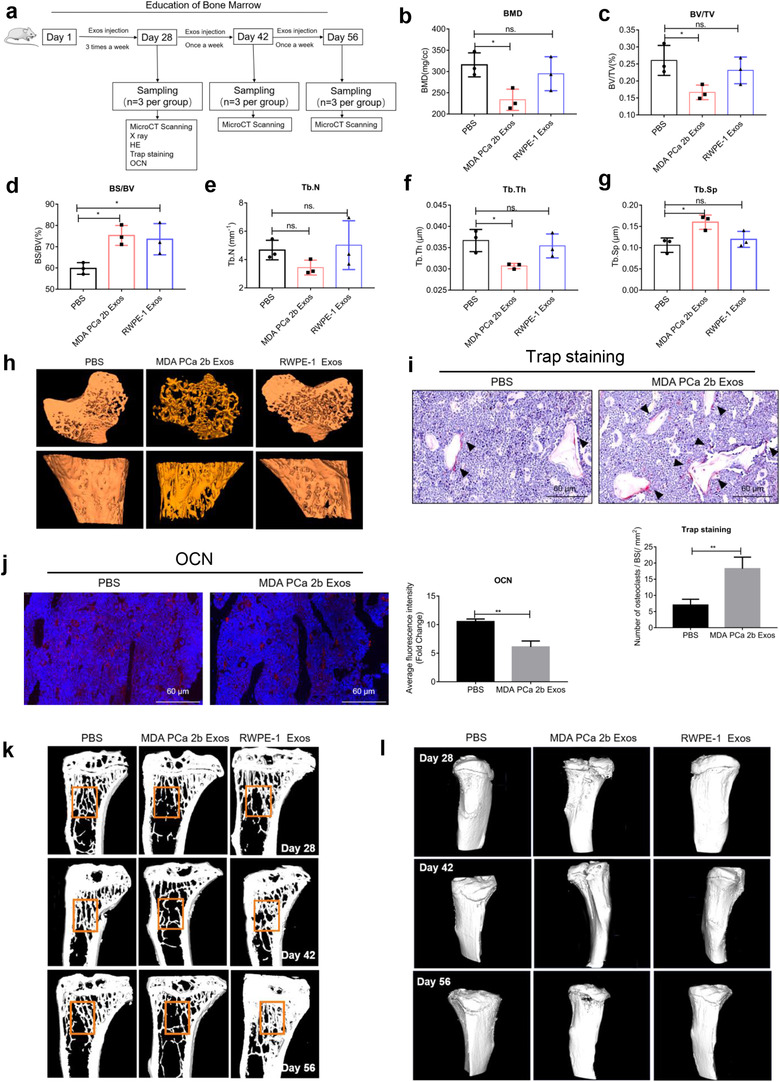
Osteogenic tumor‐derived exosomes induce osteolysis in vivo. (a) Experimental design. (b–g) After 4 weeks of injection of PBS (Control), MDA PCa 2b Exos, and RWPE‐1 Exos (10 μg, thrice weekly), micro‐CT analysis was performed to determine BMD (b), BV/TV (c), BS/BV (d), Tb.N (e), Tb.Th (f), and Tb.Sp (g) in the harvested tibia. Decreased BMD, BV/TV, Tb.Th and increased BS/BV, Tb.Sp are shown in MDA PCa 2b Exos group. (h) Representative micro‐CT 3D‐reconstruction models illustrating trabecular separation in tibia after 4 weeks of exosome injection. (i‐j) Representative Trap staining and OCN immunofluorescence labelling of sections after 4 weeks of exosome injection. Increase in osteoclasts and decrease in osteoblasts are shown. Scale bar = 60 μm. (k‐l) Representative longitudinal‐section images (k) and 3D maps (l) of the collected tibia at different time points. Bone loss in MDA PCa 2b group at each time point is shown. Data were analysed using *t* test (i‐j) and one‐way ANOVA with multiple comparisons test (b‐g). *P < 0.05; **P < 0.01; ***P < 0.001

Considering the key role of PCa exosomes in bone homeostasis in vitro, we sought to test whether PCa‐derived exosomes influence bone formation and bone lysis in vivo. We treated male BALB/C nude mice (7 weeks old, *n* = 3 per group/time point) with PBS (Control group), PCa exosomes, or normal exosomes through tail‐vein injection and then harvested the tibia for experiments (Figure 5a); we harvested the tibia after 4 weeks of injection of exosomes (10 μg in 100 μl of PBS, thrice weekly) or 100 μl of PBS alone. No differences between the groups was observed in morphological examinations of the mice and of bone or in X‐ray scanning (Figure S4a–c). The results of micro‐CT analyses showed that four measured parameters, BMD, BV/TV, Tb.N, and Tb.Th of diaphysis, from the right tibia were markedly lower in the MDA PCa 2b Exos group than in the control groups, whereas BS/BV and Tb.Sp of diaphysis were notably elevated in the MDA PCa 2b Exos group (Figure 5b‐g). These results indicated that osteolysis occurred in bone after 4‐weeks MDA PCa 2b exosomes education via tail‐vein. Representative 3D‐reconstruction models of the separated trabecular bones are shown in Figure 5h. At sacrifice, the left tibia was collected for H&E and TRAP staining and OCN immunofluorescence labelling. Increased severity of bone destruction in the MDA PCa 2b Exos group was indicated by H&E staining results (Figure S4d), and increased numbers of osteoclasts with fewer osteoblasts were indicated by the results of TRAP staining (Figure 5i) and OCN immunolabeling (Figure 5j). By contrast, H&E staining of soft tissues revealed no overt tissue damage, which confirmed the bone‐specific functional targeting of the PCa exosomes (Figure S4e).

After 4 weeks of injection, it became challenging to further inject the mouse tails with exosomes. Therefore, we altered our injection strategy to a single injection per week of a larger dose of exosomes (50 μg of exosomes in 150 μl of PBS, with 150 μl of PBS alone used as a control), and we harvested the tibia on Days 42 and 56 and performed micro‐CT analysis; representative longitudinal‐section images and 3D maps of the tibia are shown in Figure 5k,l, respectively. Bone loss was detected in the MDA PCa 2b Exos group at all three indicated time points as compared with both the Control group and the RWPE‐1 Exos group.

Next, we educated the bone marrow of BALB/C nude mice (7 weeks old, *n* = 6 per group) with C4‐2 and PC3 exosomes for 4 weeks and then harvested the right tibia and performed micro‐CT analysis. Osteolysis was verified based on the bone parameters BMD, BV/TV, BS/BV, and Tb.Th (Figure S5a–d), although Tb.N and Tb.Sp did not differ between the groups (Figure S5e,f). Representative 3D‐reconstruction models are shown in Figure S5g. Increase in osteoclasts was indicated by Trap staining images and quantification (Figure S5h‐j).

Collectively, these results confirmed that a small‐dose injection of PCa exosomes derived from osteogenic, osteoclastic, or mixed PCa cells (without the help of tumor cells) can markedly induce osteolysis and decrease bone formation in model mice; this agrees with our in vitro findings and is the opposite of what was previously accepted.

### 3.6 PCa exosomes accelerate tumor growth in bone

**FIGURE 6:**
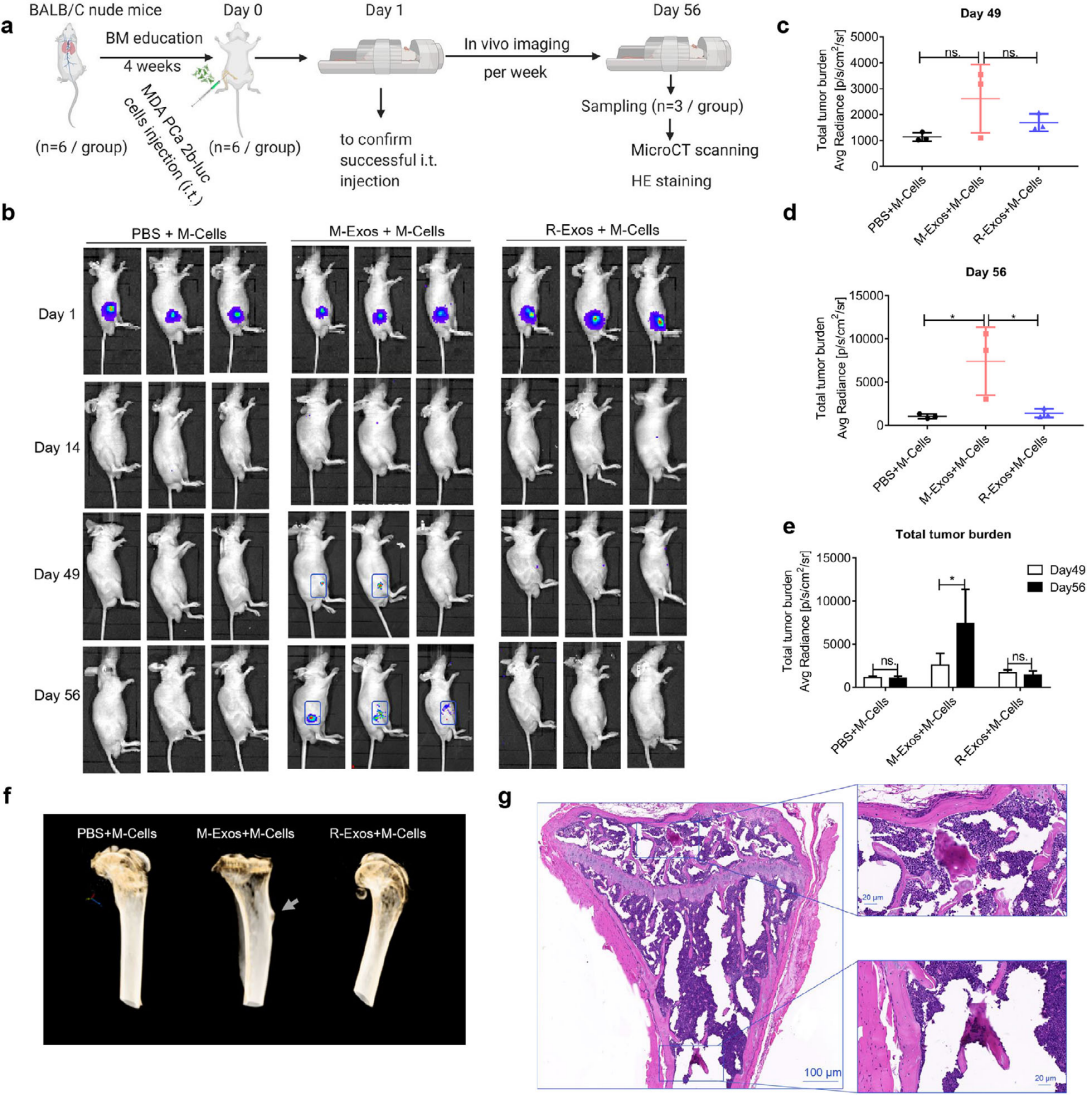
PCa exosomes accelerate tumor cell growth in bone. (a) Experimental design. (b) In vivo imaging of BALB/C nude mice pretreated with PBS, MDA PCa 2b Exos, or RWPE‐1 Exos for 28 days and then administered an i.t. injection of MDA‐PCa‐2b‐luc cells. Tumor growth is shown only in M‐Exos+M‐Cells group at Days 49 and 56 by in vivo imaging. (c–d) Quantification of total tumor burden on Day 49 (c) and Day 56 (d) between groups. Total tumor burden is shown to increase dramatically in M‐Exos+M‐Cells group. (e) Comparison of total tumor burden within groups at different time points. Tumor growth in bone was accelerated only in M‐Exos+M‐Cells group. (f) Representative 3D‐reconstruction images from micro‐CT analysis. Osteoblastic lesions were observed only in M‐Exos+M‐Cells group. (g) Representative H&E staining of tumor in bone of M‐Exos+M‐Cells group on Day 56. Data were analysed using one‐way ANOVA with multiple comparisons test (c‐d) and *t* test (e). *P < 0.05; **P < 0.01; ***P < 0.001

After successful bone marrow education and induction of osteoclastic lesions in BALB/C nude mice, we aimed to determine, based on our previous results, whether PCa exosomes can accelerate tumor growth in bone. After 4 weeks of bone marrow education, 2.5 × 10^6^ MDA PCa 2b cells infected with a lentivirus vector (GFP‐Puro‐Luc) were injected into the right tibia of BALB/C nude mice (*n* = 6 per group, start time point) (Figure 6a), and then in vivo imaging was performed from Day 1 to Day 56 post‐injection; the imaging on Day 1 was performed to ensure that the i.t. injection was successful and that the bioluminescence intensity did not differ between the different groups (Figure 6b). During Days 14–42, no bioluminescence was detectable in any of the groups, probably due to immune elimination, but then on Days 49 and 56 (*n* = 3 per group, end time point, three mice in the M‐Exos+M‐Cells group were dead before Day 49), bioluminescence was detectable only in the MDA PCa 2b group, which indicates that pre‐education with tumor exosomes accelerated tumor growth in bone (Figure 6b). On Days 49 and 56, the tumor burden in the different groups was quantified through in vivo imaging. Bioluminescence was detected both on Day 49 (Figure 6c) and Day 56 (Figure 6d), and the bioluminescence intensity was increased drastically (Figure 6e) in the M‐Exos+M‐Cells group, which indicated that the growth of the tumor cells was accelerated. When the mice of the MDA Exos group were sacrificed on Day 56, bone tumor was detected in 3D‐reconstruction images from micro‐CT analysis (Figure 6f), and H&E staining revealed the presence of prostatic tumor in bone (Figure 6g).

### 3.7 miRNAs transferred by MDA PCa 2b exosomes influence bone homeostasis

**FIGURE 7:**
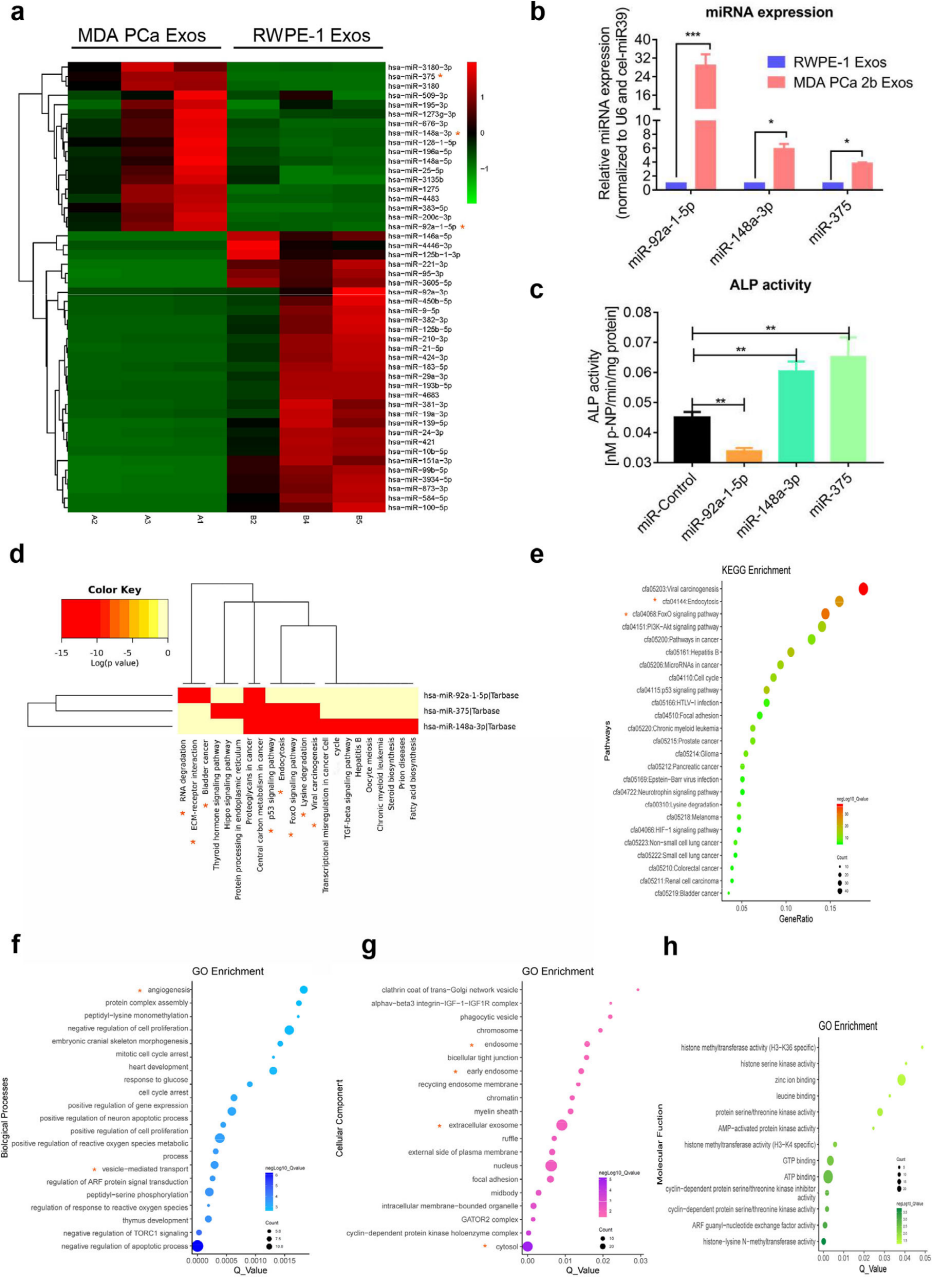
miRNAs transferred by PCa exosomes regulate bone homeostasis. (a) Heatmap of miRNAs differentially expressed between RWPE‐1 Exos and MDA PCa 2b Exos. Red: increased expression; green: decreased expression. (b) High expression of miR‐92a‐1‐5p, miR‐148a‐3p, and miR‐375 in MDA PCa 2b exosomes was confirmed using qPCR. U6 RNA and cel‐miR‐39: internal and external controls. (c) ALP activity was determined after overexpression of miR‐92a‐1‐5p, miR‐148a‐3p, and miR‐375. Decreased and increased ALP activity are shown in miR‐92a‐1‐5p group and miR‐148a‐3p/miR‐375 group, respectively. (d) KEGG enrichment analysed using DIANA tool. (e) Bubble plot showing KEGG enrichment by mRNAs targeted in functional pathways. (f) Bubble plot showing GO enrichment in Biological Process. (g) Bubble plot showing GO enrichment in Cellular Component. (h) Bubble plot showing GO enrichment in Molecular Function. Data were analysed using *t* test (b) and one‐way ANOVA with multiple comparison test (c). *P < 0.05; **P < 0.01; ***P < 0.001

The results obtained thus far led us to investigate whether exosome cargos influence bone homeostasis and tumor development. Among the exosome cargos, miRNA have been studied most thoroughly and are highly conserved among species; they are small but powerful RNA molecules with multiple target genes that have been demonstrated to be critical regulator of multiple diseases. Therefore, we used next‐generation sequencing to profile small RNAs from MDA PCa 2b exosomes and RWPE‐1 exosomes, and we screened for differentially expressed miRNAs by using these criteria: |log2(FC)| > 2.5 (FC: fold‐change) and *P* < 0.001. Among the miRNA identified to be differentially expressed, 18 were upregulated and 30 were downregulated in MDA PCa 2b exosomes relative to controls (Figure 7a). Next, we focused on three specific miRNAs, miR‐92a‐1‐5p, miR‐375, and miR‐148a‐3p. miR‐92a‐1‐5, a newly identified and seldom reported member of the miR‐17‐92‐cluster miRNAs, has been shown to be associated with cancer (Liu et al., 2019); it was reported to be markedly downregulated in exosomes isolated from the urine of PCa patients and could serve as a potential noninvasive biomarker of PCa (Rodríguez et al., 2017). Our previous work demonstrated that urinary exosomal miR‐375‐3p is a potential biomarker that exhibits strong association with PCa clinical stage and bone metastasis (Li et al., 2019). miR‐148a‐3p was reported to be upregulated in the serum of PCa patients (Dybos et al., 2018). The results of qPCR analyses confirmed that miR‐92a‐1‐5p, miR‐375, and miR‐148a‐3p were expressed at higher levels in MDA PCa 2b exosomes than in RWPE‐1 exosomes (Figure 7b), and miR‐92a‐1‐5p was the most abundant miRNA among eight miRNAs verified to be upregulated by using qPCR (data not shown). To examine the potential roles of the three miRNAs in bone homeostasis, we first transfected MC3T3‐E1 cells with the miRNAs for 7 days; after the induction, ALP activity was measured, which revealed that miR‐92a‐1‐5p overexpression inhibited the osteogenic differentiation of MC3T3‐E1 cells, whereas overexpression of miR‐148a‐3p or miR‐375 enhanced osteogenic differentiation (Figure 7c).

Next, we used the tool DIANA to perform KEGG analyses for the target genes of the three miRNAs, and we identified a total of 22 pathways (*P* < 0.05). Considering the distinct roles of the three miRNAs in bone homeostasis, for further investigation, we selected these eight potential functional pathways: RNA degradation, Extracellular matrix (ECM)‐receptor interaction, Bladder cancer, p53 signalling pathway, Endocytosis, FOXO signalling pathway, Lysine degradation, and Viral carcinogenesis (Figure 7d). Our results identified 154 genes in the eight pathways as potential target genes of the three miRNAs. Moreover, to reveal the potential functions of these target genes, we performed KEGG and GO enrichment analyses by using the tool DAVID; the target genes were enriched in FOXO signalling pathway, angiogenesis, exosome biosynthesis, cytosis, and cancer‐associated pathways (*P* < 0.05) (Figure 7e–h). Notably, FoxOs (FoxO1, FoxO3, and FoxO4), which are expressed in numerous types of bone cells, have been widely shown to activate osteoblastogenesis and suppress osteoclastogenesis through several targets and pathways (Ma et al., 2020), and bone tissue regeneration is frequently linked with angiogenesis and cytosis. These results indicated that miRNAs delivered by PCa exosomes strongly and broadly affect bone homeostasis through multiple potential pathways and target genes.

### 3.8 miR‐92a‐1‐5p transferred by MDA PCa 2b exosomes facilitates osteoclast differentiation and inhibits osteoblast differentiation by targeting COL1A1

**FIGURE 8:**
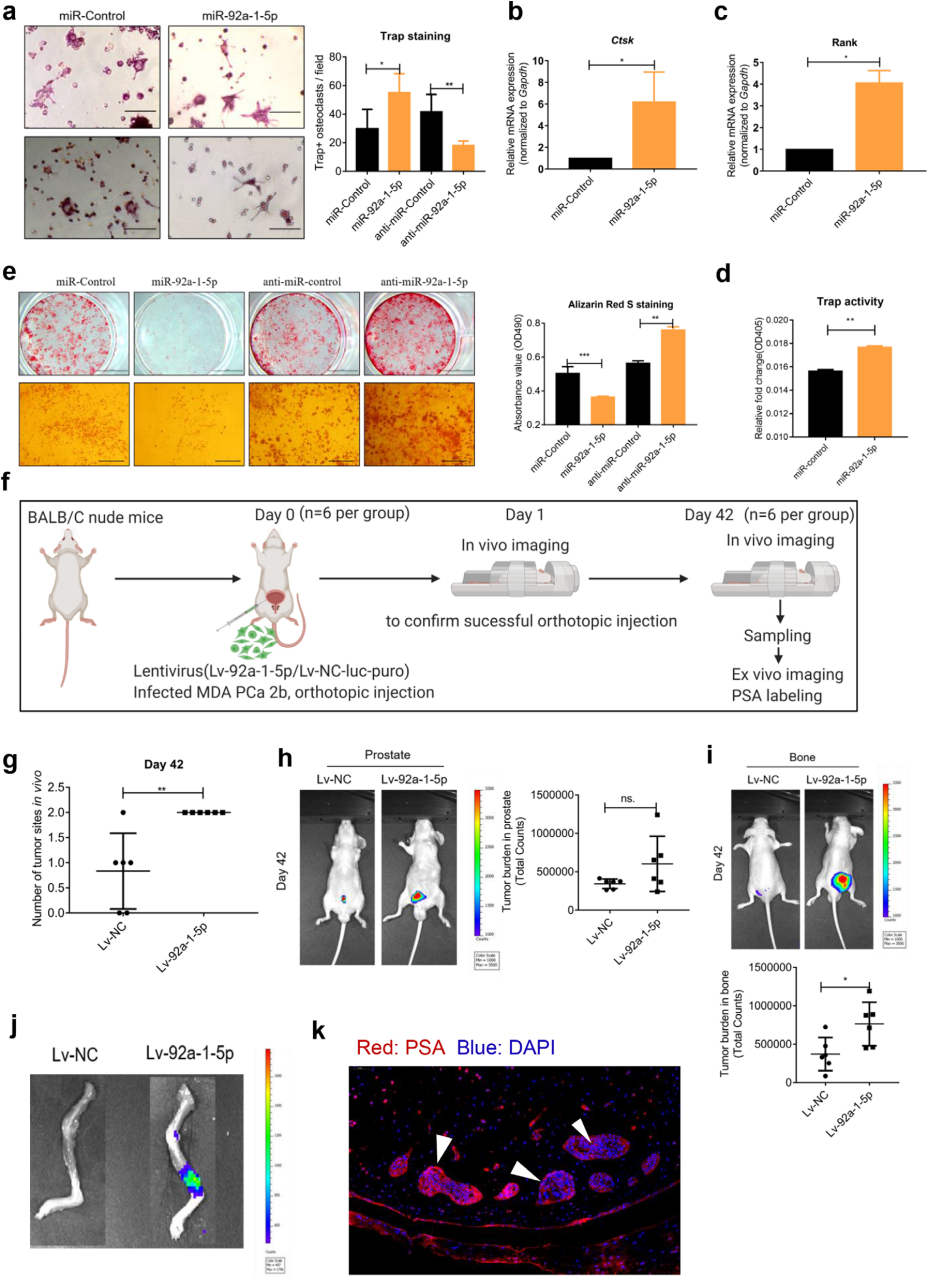
miR‐92a‐1‐5p induces osteoclast differentiation, inhibits osteoblastogenesis, and promotes prostate cancer bone metastasis. (a) Representative Trap‐staining images and quantification. Scale bar = 100 μm. (b–c) mRNA expression of Ctsk and Rank was elevated after transient overexpression of miR‐92a‐1‐5p. (d) Trap activity was increased in Raw264.7 cells after treatment with 100 μM miRNA for 6 days. (e) Alizarin Red S staining of MC3T3‐E1 cells at Day 21 after transfection with indicated miRNAs in osteogenic induction medium. Scale bars = 5 μm (upper) and 100 μm (lower). (f) Experimental design for miR‐92a‐1‐5p analysis in vivo. (g) Examination of total tumor burden. Number of tumor sites was markedly higher in Lv‐92a‐1‐5p group. (h) Tumor burden in prostate on Day 42. (i) Tumor burden in bone was higher in Lv‐92a‐1‐5p group. (j) Confirmation of tumor bone metastasis by ex vivo imaging. (k) Confirmation of tumor bone metastasis by PSA immunofluorescence labelling. Data were analysed using *t* test. *P < 0.05; **P < 0.01; ***P < 0.001

Lastly, we investigated the precise role in bone homeostasis and tumor development of miR‐92a‐1‐5p, which was the most abundant miRNA that we detected here, and which has been seldom examined in previous studies. To determine the function of miR‐92a‐1‐5p during osteoclast differentiation, we transfected Raw264.7 cells with miRNA mimics or inhibitors, and after culturing the cells with RANKL for 6 days, we used Trap staining to assess osteoclast formation (Figure 8a). Moreover, we quantified the mRNA expression of the osteoclast markers Ctsk and Rank and measured Trap activity (Figure 8b‐d). Overall, osteoclast differentiation was increased by miR‐92a‐1‐5p and inhibited by anti‐miR‐92a‐1‐5p. Furthermore, to elucidate the role of miR‐92a‐1‐5p during osteoblast differentiation, we transfected MC3T3‐E1 cells with the miRNA mimics and inhibitors, and after induction for 21 days, performed Alizarin Red S staining. Osteoblast differentiation was decreased by the miRNA mimics and increased by the miRNA inhibitors (Figure 8e). Next, we injected cells stably transfected with a miRNA‐expressing lentivirus (miR‐92a‐1‐5p MDA‐PCa‐2b‐luc cells) into the prostate of BALB/C nude mice (7 weeks old, *n* = 6 per group) (Figure 8f) and then quantified the tumor burden through in vivo imaging and ex vivo imaging on Day 42. Total tumor burden was higher in Lv‐92a‐1‐5p group via observing the number of tumor sites (Figure 8g). Elevated tumor burden was observed in bone (Figure 8i) in Lv‐92a‐1‐5p group, however, tumor burden in prostate was not significantly increased (Figure 8h). Tumor bone metastasis was confirmed via ex vivo imaging (Figure 8j) and PSA immunofluorescence labelling (red, white arrows indicated) (Figure 8k), based on PSA expression in prostate and MDA PCa cells, and no expression in bone.

**FIGURE 9:**
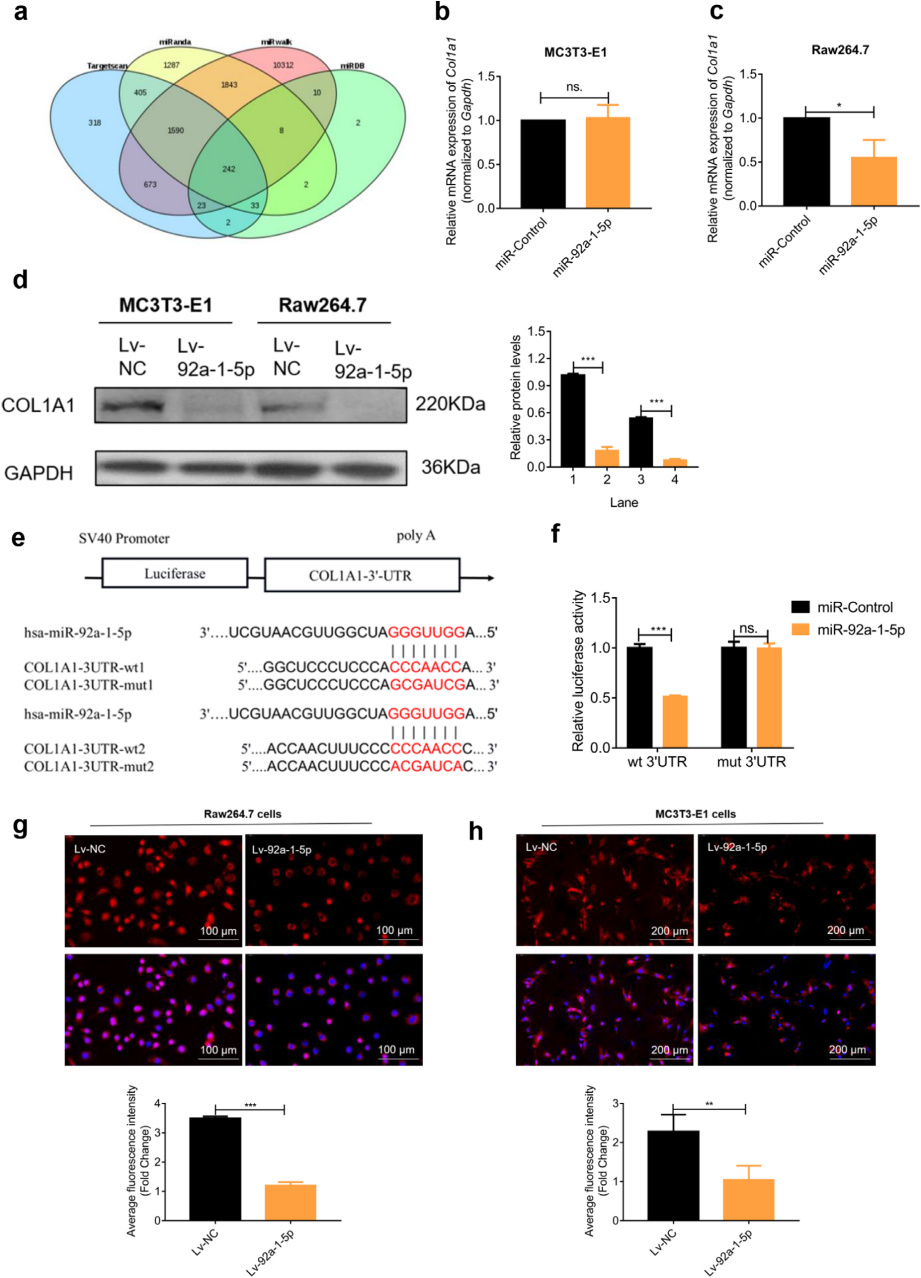
miR‐92a‐1‐5p targets *COL1A1* and type I collagen. (a) Predicted targets of miR‐92a‐1–5p identified using four independent platforms. (b–c) COL1A1 mRNA level determined using qPCR after 48 h of transfection. (d) COL1A1 protein expression in cells stably transfected with lentivirus. GAPDH: internal control. (e) Schematic of vectors used. (f) Relative luciferase activity determined after 48 h of co‐transfection. (g‐h) Immunofluorescence labelling of type I collagen in cells stably transfected with lentivirus. Marked decrease in average fluorescence intensity is shown in Raw264.7 cells and MC3T3‐E1 cells. Data were analysed using *t* test. *P < 0.05; **P < 0.01; ***P < 0.001

To uncover the molecular mechanisms underlying miR‐92a‐1‐5p‐regulated bone homeostasis, we first used the tool DIANA to identify enriched KEGG pathways. Among the enriched pathways, the most dominant was ECM‐receptor interaction (Table 1). Next, we predicted target genes by using online miRNA prediction software (Targetscan, miRanda, miRWalk, and miRDB) (Figure 9a). Among the predicted targets, we identified *COL1A1*, which encodes a type I collagen subunit that is the major organic component of the bone ECM (Kolb & Bussard, 2019). At 48 h after transfection with miR‐92a‐1‐5p, the mRNA level of *COL1A1* was not notably altered in MC3T3‐E1 cells but was significantly decreased in Raw264.7 cells (Figure 9b,c). Western blotting results showed that COL1A1 protein levels were decreased in MC3T3‐E1 and Raw264.7 cells stably transfected with the miR‐92a‐1‐5p lentivirus (Figure 9d). Moreover, according to the results of bioinformatics analysis, *COL1A1* harboured two 7‐nt seed sites for miR‐92a‐1‐5p within its 3ʹ‐UTR (Figure 9e). Thus, we used a dual‐luciferase reporter‐gene system to determine whether miR‐92a‐1‐5p directly targets *COL1A1*; luciferase activity in the miR‐92a‐1‐5p and *COL1A1*‐Wt co‐transfection group was significantly lower than that in the control group (*P* < 0.001) (Figure 9f), which implied that *COL1A1* is a direct target of miR‐92a‐1‐5p. We further confirmed these results by using immunofluorescence labelling to examine the cellular localization and relative expression level of type I collagen in MC3T3‐E1 and Raw264.7 cells stably expressing the miR‐92a‐1‐5p lentivirus. As expectedly, the expression levels of type I collagen in MC3T3‐E1 and Raw264.7 cells were dramatically decreased (Figure 9g,h).

**TABLE 1:** Enriched KEGG pathways identified using DIANA miRPath V3.0 (*P* < 0.05)

KEGG pathway	*P*‐value	Genes
1.ECM‐receptor interaction	6.99E‐12	LAMB2;THBS1;THBS2;COL4A5;SV2A;ITGAV;COL4A2;DAG1
2.Central carbon metabolism in cancer	8.95E‐05	FGFR3;GCK;PKM;MYC;PFKL;FGFR1;MAPK1;SLC1A5
3.Other glycan degradation	0.006624	NEU3
4.Proteoglycans in cancer	0.007546	THBS1;CAV1;PTK2;ITGAV;MMP2;MYC;TIMP3;DDX5;LUM;CDKN1A;FGFR1;MAPK1
5.RNA degradation	0.011392	CNOT6;TOB2;DDX6;ENO1;PABPC1;CNOT1;PFKL;ENO2
6.Bacterial invasion of epithelial cells	0.023308	CRKL;SHC1;CAV1;BCAR1;VCL;PTK2;ARPC2;SEPT3
7.Regulation of actin cytoskeleton	0.033455	SSH2;FGFR3;CRKL;SSH1;CFL1;BCAR1;VCL;PTK2;ITGAV;ARPC2;PIP5K1A;ACTN4;FGFR1;MAPK1
8.Bladder cancer	0.033455	FGFR3;THBS1;MMP2;MYC;CDKN1A;MAPK1
9.Focal adhesion	0.044213	LAMB2;CRKL;SHC1;THBS1;THBS2;CAV1;COL4A5;BCAR1;VCL;PTK2;ITGAV;COL4A2;ACTN4;MAPK1;ARHGAP5

**FIGURE 10:**
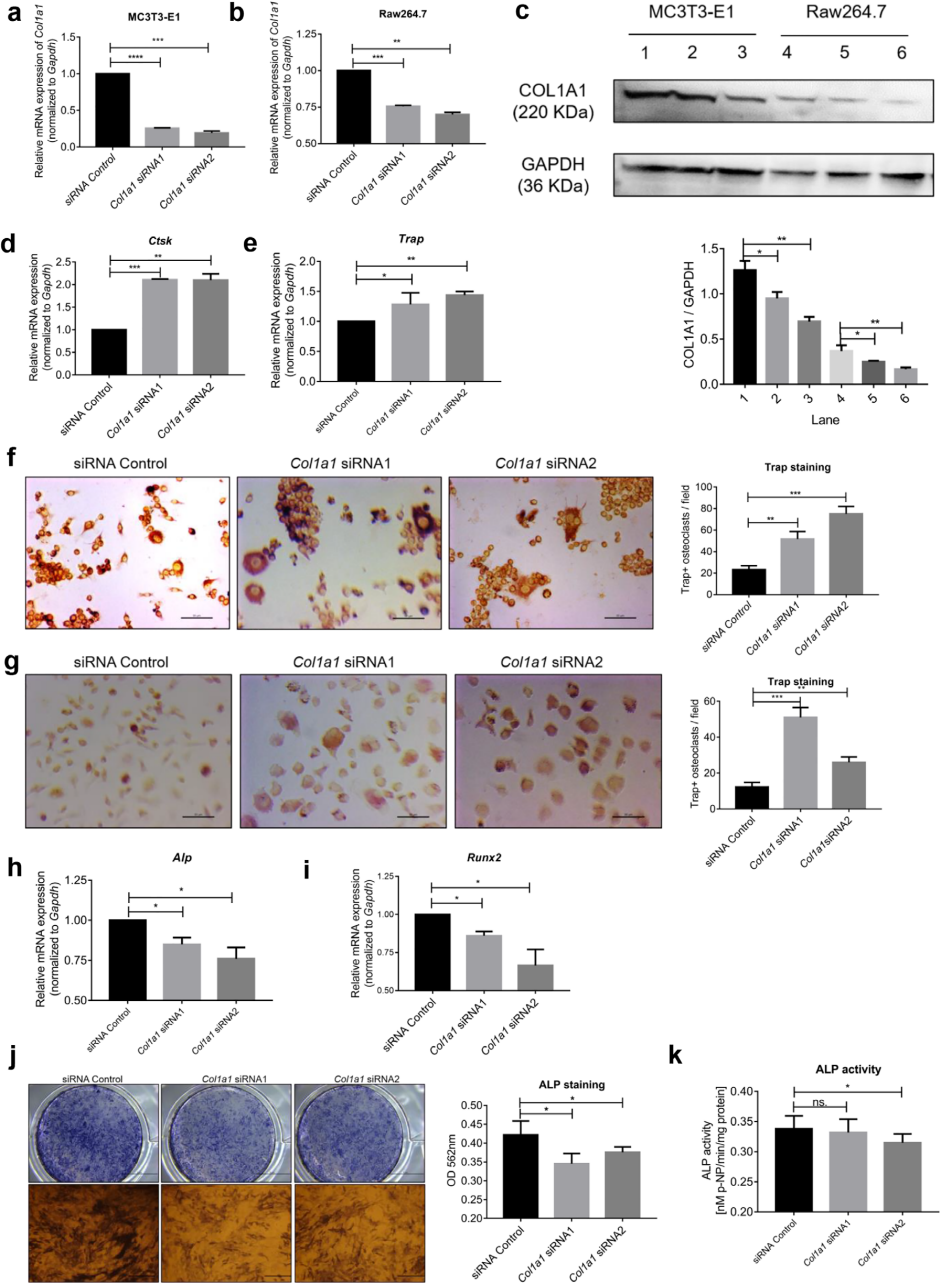
Effects of COL1A1 knockdown on osteoclast and osteoblast differentiation. (a) qPCR analysis of mRNA expression of Col1a1 in MC3T3‐E1 cells transfected with indicated siRNAs and siRNA Control. (b) qPCR analysis of mRNA expression of Col1a1 in Raw264.7 cells transfected with indicated siRNAs and siRNA Control after 36 hours. (c) Protein expression of COL1A1 in MC3T3‐E1 cells and Raw264.7 cells transfected with indicated siRNAs and siRNA control after 48 h. Lane 1,4: siRNA Control; Lane 2,5: Col1a1 siRNA1; Lane 3,6: Col1a1 siRNA2. (d‐e) Upregulation of Ctsk and Trap mRNA expression in Raw264.7 cells as assessed by qPCR 36 h post‐transfection. (f‐g) Representative Trap‐staining image (left, scale bar = 50 μm) and quantification (right) of Trap^+^ osteoclasts in Raw264.7 cells and BMMs. Increase in Trap^+^ osteoclasts is shown. (h‐i) Downregulation of Alp and Runx2 mRNA expression in MC3T3‐E1 cells by qPCR 36 h post‐transfection. (j) ALP staining of MC3T3‐E1 cells 7 days after transfection. Reduced ALP staining is shown. (k) ALP activity of MC3T3‐E1 cells 7 days after transfection. Reduced ALP activity is shown. Data were analysed using one‐way ANOVA with multiple comparisons test. *P < 0.05; **P < 0.01; ***P < 0.001

To further demonstrate the effect of *COL1A1* knockdown in the differentiation of osteoblasts and osteoclasts, we designed and transfected *Col1a1* siRNA, and confirmed the decrease of COL1A1 expression in both MC3T3‐E1 cells and Raw264.7 cells using qPCR (Figure 10a,b) and western blotting (Figure 10c). As expected, the mRNA expression of Ctsk and Trap in Raw264.7 cells was upregulated following transfection with *Col1a1* siRNA for 36 h (Figure 10d,e); the number of Trap^+^ osteoclasts were confirmed increase in both Raw264.7 cells (Figure 10f) and BMMs (Figure 10g) 6 days after transfection. Moreover, Alp and Runx2 mRNA (Figure 10h,i) expression in MC3T3‐E1 cells was downregulated 36 h post transfection; ALP staining (Figure 10j) and ALP activity (Figure 10k) were also reduced after transfection for 7 days.

**FIGURE 11:**
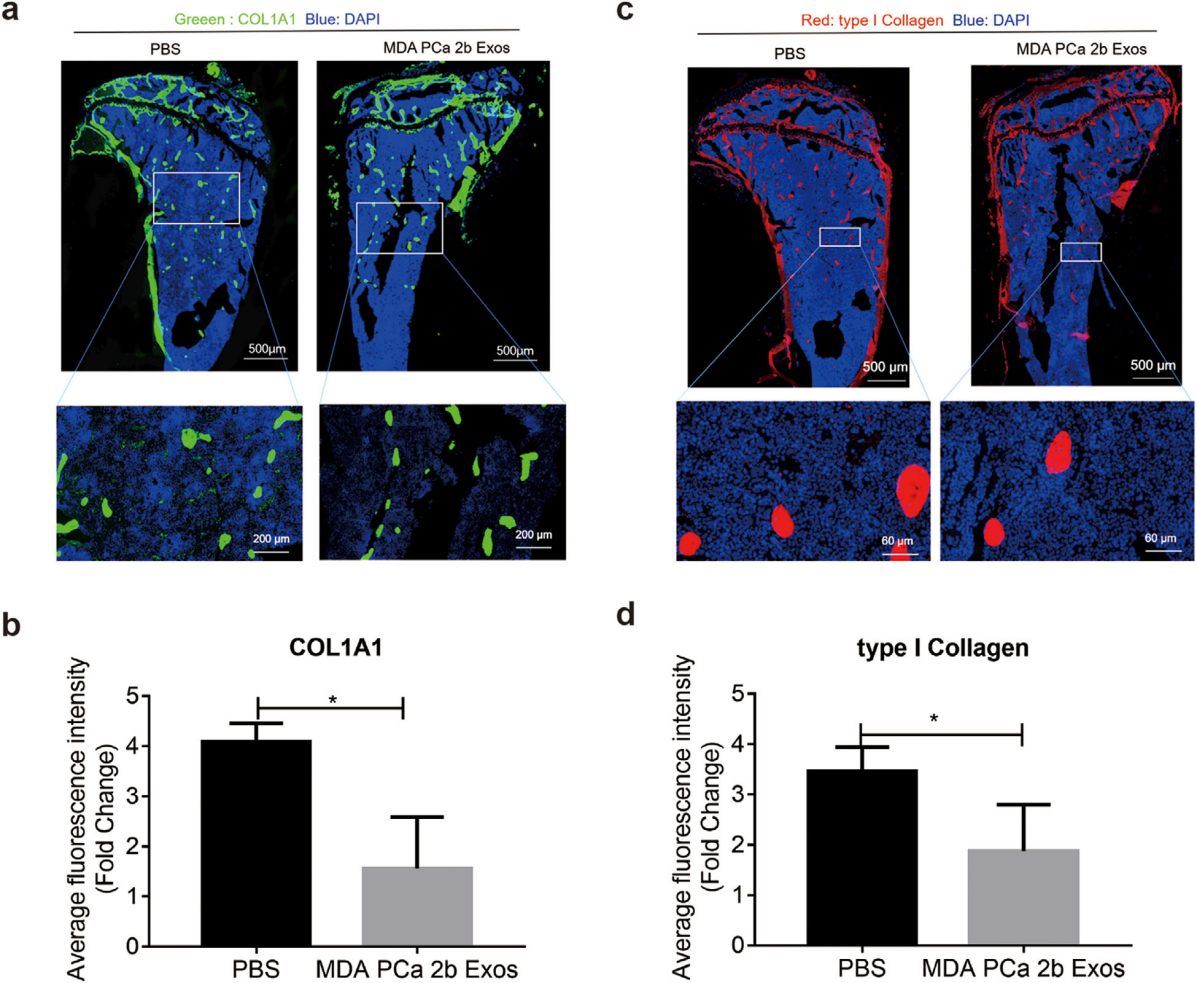
MDA PCa 2b exosomes containing miR‐92a‐1‐5p degrade COL1A1 and type I collagen in vivo. (a) Immunofluorescence labelling of COL1A1 from bone of BALB/C nude mice educated with MDA PCa 2b exosomes for 4 weeks. Markedly decrease expression of COL1A1 in bone is shown. (b) Quantification analysis of COL1A1 expression after 4‐weeks exosomes education. (c) Immunofluorescence labelling of type I collagen from bone of BALB/C nude mice educated with MDA PCa 2b exosomes for 4 weeks. (d) Quantification analysis of type I collagen expression after 4‐weeks exosomes education. Data were analysed using *t* test. *P < 0.05; **P < 0.01; ***P < 0.001

To test whether exosomes from MDA PCa 2b cells can diminish COL1A1 expression in vivo, we injected nude mice thrice weekly for 4 weeks with 10 μg of MDA PCa 2b exosomes and then harvested the tibia and examined COL1A1 levels (Figure 11a,b). COL1A1 expression was clearly downregulated. We also measured the level of type I collagen in the tibia harvested after 4 weeks of bone marrow education, and we found that the type I collagen labelling intensity was lower in the MDA PCa 2b treatment group than in the control group (Figure 11c,d). We found that both COL1A1 and type I collagen mainly localize in trabecular bone. However, as mentioned in Figure 5 and Figure S5, 4‐weeks exosomes education decreased the thickness of trabecular bone and increased the trabecular space; thus, these results are consistent with the results we described in the previous part of this study.

**FIGURE 12:**
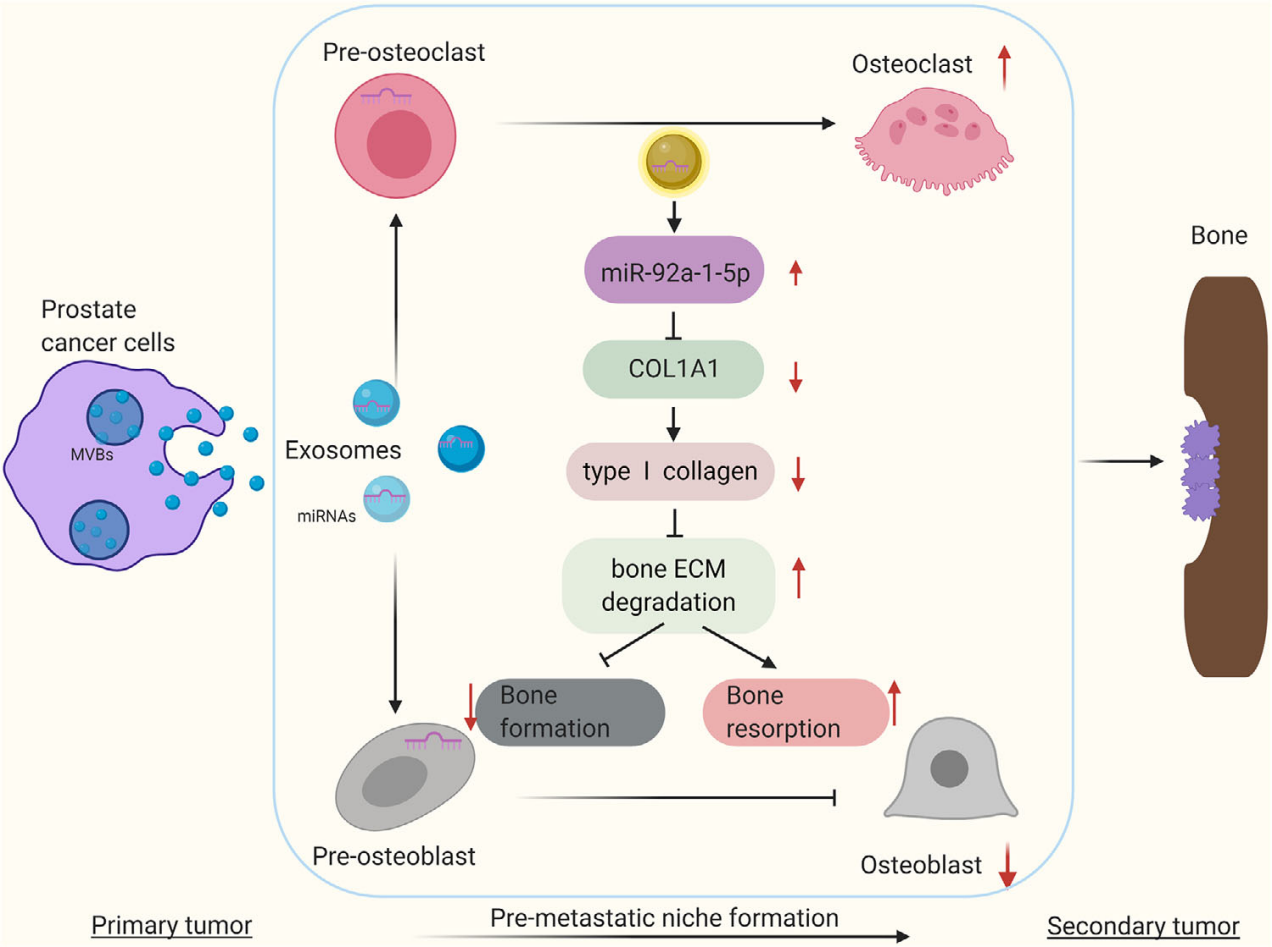
Schematic depiction of our work. PCa exosomes regulate bone homeostasis, bone ECM remodeling, and formation of a pre‐metastatic niche by transferring miR‐92a‐1‐5p and targeting *COL1A1*.

In conclusion, these results demonstrated that miR‐92a‐1‐5p derived from PCa exosomes disrupts bone homeostasis, promotes osteoclast differentiation, inhibits osteoblast differentiation, and promotes tumor bone metastasis in PCa; moreover, our findings suggest the following underlying mechanism: miR‐92a‐1‐5p and MDA PCa exosomes containing miR‐92a‐1‐5p target *COL1A1* and accelerate bone ECM degradation.

### 3.9 Reasons for discrepancy of PCa cells and PCa exosomes on bone homeostasis

Notably, unlike PCa cells, exosomes from three cell lines used in the present study (osteoblastic MDA PCa 2b cells, osteoclastic PC3 cells, and mixed C4‐2) all promoted osteoclast differentiation. Although, there may be other factors responsible for this observation, we attempted to investigate this discrepancy from the perspective of this study. Firstly, we performed qPCR to examine the expression of miRNA‐92a‐1‐5p, miRNA‐375, miR‐148a‐3p in different PCa cells. We found all three miRNAs were universally expressed, but at different level, in the three PCa cell lines (Figure S7a‐c). Based on previous data in Figure 7, miRNA‐92a‐1‐5p is osteoclastic and miRNA‐375/miR‐148a‐3p are osteoblastic. We calculated the relative osteogenic index as: relative osteogenic index = relative miRNA expression of (miR‐148a‐3p + miR‐375 ‐ miR‐92a‐1‐5p) (Figure S7d). We found that PC3 cells showed lower osteogenic index, which may partly account for PC3 as an osteoclastic cell line. Furthermore, we compared the exosome yield of the three types of PCa cell lines and found no differences (Figure S7e). We also injected BALB/C nude mice (7 weeks old, *n* = 3 per group) with 100 μg Vybrant DID labelled exosomes via tail vein and compared the fluorescence intensity in bone 24 h post injection using ex vivo imaging. No distribution difference was discovered among the groups (Figure S7f). Subsequently, we incubated 10 μg Vybrant DID labelled PCa exosomes with Raw264.7 cells and MC3T3‐E1 cells in vitro for 90 min and 180 min, respectively (Figure S7g,h). We discovered that PC3 exosomes were more easily internalized by Raw264.7 and C4‐2 exosomes were more easily internalized by MC3T3‐E1, which indicated that different PCa exosomes may target different recipient cells to play the same role. Thus, we concluded that miRNA expression in the PCa cells may be the determinants of the osteogenic characteristics of PCa cells; PCa exosomes target different recipient cells but play the same role in bone homeostasis. Importantly, the similar role, PCa exosomes derived from different types of PCa cells played in bone homeostasis, has created the basis for PCa‐exosome‐targeted therapeutic strategies for bone diseases.

## 4 DISCUSSION

PCa bone metastasis is the result of the crosstalk between tumor cells and the target bone; however, how tumor cells interact with the bone microenvironment through exosomes is poorly understood, and urgent demand exists for therapies that can enhance the survival of PCa patients with bone metastasis. Here, we used exosomes derived from osteoblastic (MDA PCa 2b), osteoclastic (PC3), and mixed (C4‐2) PCa cells to investigate how PCa exosomes influence the differentiation of both osteoblasts and osteoclasts. We showed that PCa exosomes, which were found here to promote osteoclast differentiation and concurrently inhibit osteoblastogenesis in vitro and in vivo, are responsible for osteolytic lesions, bone ECM remodeling, and the aggressive growth of PCa cells in bone. We further discovered that miRNAs selectively enriched in PCa exosomes play critical roles in bone homeostasis.

Unlike the bone metastasis of breast cancer or lung cancer, PCa bone metastasis has been uniquely defined as being “osteoblastic” based on plain radiographs, bone scans, and increased serum ALP expression. However, clinically, PCa patients are frequently reported with bone loss and subsequent fracture. Moreover, the N‐telopeptide of type I collagen (Ntx), a highly specific osteolysis biomarker, is overexpressed in patients with PCa bone metastasis (Coleman et al., 2005). Histological analyses of bone lesions in PCa bone metastasis have shown that both osteopenic and osteodense lesions exist in “osteoblastic” lesions (Roudier et al., 2008), and available treatment options for patients diagnosed with PCa bone metastasis mainly include bisphosphonates and anti‐RANKL therapies, both of which are potent and specific bone‐resorption inhibitors. However, most of the current studies on PCa bone metastasis are focused on the osteoblastic activity of osteoblasts and ignore the key underlying roles in bone resorption of osteoclasts. Here, by concomitantly examining the differentiation of osteoblasts and osteoclasts, we demonstrated that PCa exosomes create an osteoclastic premetastatic niche for metastatic PCa cells. Our findings agree with a series of previous studies indicating that bone resorption occurs before any invasion in “osteoblastic” or “osteoclastic” bone lesions (Jacobs, 1983; Sottnik & Keller, 2013; Suva et al., 2011). Additionally, our present study provides novel insights into bone homeostasis that underlies the progression of PCa bone metastasis and may be useful for the optimization of therapeutic interventions. Currently, the main treatment strategies against PCa bone metastases are focused on breaking the “vicious osteolytic cycle.” We found that PCa exosomes can increase osteoclast differentiation without the help of RANKL (Figure 3e,f,i, and Figure S2f), which indicates that the underlying mechanism is RANKL independent. Thus, we can potentially identify previously unknown mechanisms of osteoclast differentiation and novel therapeutic strategies for bone metastasis based on PCa exosomes. Obviously, further studies must be conducted to investigate the RANKL‐independent mechanism and relevant therapeutic targets.

The occurrence and development of bone metastasis constitute a complex process. The classical “seed and soil” hypothesis proposed by Stephen Paget in 1889 posits that tumor metastasis involves a range of specific interactions between tumor cells and resident stromal cells (Paget, 1989). Another illustration of the complex mechanism of PCa metastasis to bone is the aforementioned “vicious cycle,” in which the activity of osteoblasts and osteoclasts in the bone microenvironment is enhanced by cytokines secreted from metastatic PCa cells, and the factors secreted by osteoblasts and osteoclasts, in turn, promote the release of factors necessary for tumor growth in bone (Cook et al., 2014). Tumor exosomes can mediate crosstalk between tumours and target organs and thereby increase metastasis (Peinado et al., 2012; Rodrigues et al., 2019). With respect to PCa, potential exosomal biomarkers derived from serum or urine for diagnosis have been widely investigated (Cho et al., 2019; Hao et al., 2015). Interestingly, recent studies showed miRNAs, with potential clinical implications for cancer therapy, are involved in EV secretion from prostate cancer (PCa) cells (Urabe et al., 2020; Urabe et al., 2020). However, only a few functional studies on extracellular vesicles have been conducted to delineate whether PCa exosomes can mechanistically influence bone metastasis. In a previous study, we verified that exosomal miR‐141‐3p derived from MDA PCa 2b cells promotes osteoblast activity and osteoblastic bone metastasis (Ye et al., 2017), and another study demonstrated that cancer‐derived hsa‐miR‐940 induces osteogenesis by targeting *ARHGAP1* and *FAM134A* (Hashimoto et al., 2018). Both studies focused on only one miRNA delivered by PCa exosomes, and the specific role of PCa exosomes was not clearly defined. Moreover, the complexity of PCa bone metastasis was underappreciated in previous studies because the detected osteoblastic lesions were overemphasized, and the underlying osteoclastic lesions were ignored. Another study showed that primary PCa educates the bone stroma through exosomal PKM2 and promotes bone metastasis (Dai et al., 2019); the study suggested a mechanism whereby primary tumor‐derived exosomes alter the bone marrow microenvironment through exosomal PKM2 and ultimately promote tumor proliferation. Here, we focused on the differentiation of bone stromal cells mediated by PCa exosomes, and our results demonstrated that PCa exosomes function as key regulators of bone homeostasis, bone remodeling, and tumor growth in bone. Thus, our study serves as a robust and highly favourable complement to the previous studies addressing the “vicious cycle” and “seed and soil” hypotheses.

Our findings enhance current understanding of how PCa exosomes induce bone lesions and how the aberrant bone affects the progression of PCa bone disease. Bone, the hardest organ in the body, is the most preferred site for metastatic PCa, but for decades, the mechanisms responsible for this have remained unclear. We observed for the first time (to our knowledge) that PCa exosomes selectively target bone stomal cells in vivo (Figure 2 and Figure S1). Although the molecules on the surface or the inside of PCa exosomes that are involved in this targeting and the precise manner in which these molecules function remain to be established, our findings provide crucial clues regarding the bone‐targeting of PCa by means of the exosomes that are secreted by the cancer cells. Further investigation is required to address this intriguing and critical question. PCa‐induced aberrant bone lesions have been confirmed to be caused by tumor‐secreted cytokines, some of which are osteoblastic and others osteoclastic. We demonstrated here for the first time (to our knowledge) that the exosomes from all three types of PCa cells are osteoclastic; this offers a new approach for designing alternative therapeutic methods based on exosomes in PCa. Our study is also the first (to our knowledge) to report that a small amount of exosomes from a solid tumor alone (without interaction with cells) can reduce bone mass in mice (Figure 5 and Figure S5), which confirms the high efficiency of the exosomes and provides a foundation for developing exosomes as therapeutic targets. However, no notable changes in bone mass was observed based on X‐ray analysis (Figure S4c). This difference is mainly due to the detection method and indicates that the method used must be highly sensitive. Overall, these findings suggest that PCa exosomes ultimately prepare a microenvironment that facilitates metastatic lesion by targeting bone stromal cells, regulating bone homeostasis, and influencing bone remodeling.

Over the past few decades, miRNAs have been recognized as critical regulators of bone metabolism. However, the functions of the miRNAs that are selectively enriched in metastatic PCa exosomes have remained incompletely elucidated. We are the first (to our knowledge) to report that two specific osteoblastic miRNAs (miR‐375 and miR‐148a‐3p) and one osteoclastic miRNA (miR‐92a‐1‐5p) coexist in PCa exosomes; this suggests that PCa exosomes can function as potential and potent modulators of bone diseases. However, as noted previously, MDA PCa 2b exosomes promote osteoclast differentiation. One explanation for this discrepancy is that only a slight upregulation (3–5‐fold) was observed in osteoblastic miR‐375 and miR‐148a‐3p as compared with the 30‐fold upregulation measured for osteoclastic miR‐92a‐1‐5p. miR‐92a‐1‐5p is a newly identified miRNA and is currently mentioned in only 16 papers found on PubMed. A previous study showed that miR‐92a‐1‐5p was downregulated during the osteoblastic differentiation of MC3T3‐E1 cells and that the miRNA affected osteogenic differentiation by negatively regulating β‐catenin (Lin et al., 2019). However, β‐catenin has not been validated as a direct target of miR‐92a‐1‐5p either experimentally or through bioinformatics analyses. Here, we demonstrated that miR‐92a‐1‐5p promoted osteoclast differentiation, concurrently suppressed osteoblast differentiation, and promoted bone metastasis in model mice. We further showed that miR‐92a‐1‐5p directly targeted the *COL1A1* 3ʹ‐UTR and thus caused type I collagen degradation. Two miRNA‐binding sites have been bioinformatically identified in the *COL1A1* 3ʹ‐UTR and vectors were designed based on the binding of both sites in the current study. However, further experiments are required to determine which of the two sites exert the binding effect.

Tumor exosomes typically trigger multiple signalling pathways, and we found that MDA PCa 2b exosomes induced drastic degradation of COL1A1 and Type I collagen in the bone ECM after bone marrow education through tail‐vein injection of the exosomes for 4 weeks. The ECM plays a crucial and dynamic role in the regulation of the tumor microenvironment (Mohan et al., 2020), and the crosstalk between bone ECM and bone metastatic cancer cells plays a critical role in regulating cancer cell growth in bone (Kolb & Bussard, 2019). Type I collagen is typical collagen triple‐helix molecule containing two α1 chains and one α2 chain and is distributed around bone cells (osteoblasts, osteoclasts, osteocytes, etc.), and type I collagen is the most abundant organic component of the bone matrix (Kolb & Bussard, 2019). PCa bone metastasis was previously shown to induce the destruction of collagen alignment in long bones (Sekita et al., 2017), although the mechanism involved has not yet been clarified. Here, we are the first (to our knowledge) to reveal that PCa exosomes facilitate the degradation of bone ECM and the dysregulation of ECM remodeling by delivering their cargo miR‐92a‐1‐5p. Furthermore, exosomes can also act as a storehouse of tissue‐remodeling proteases: Proteomic analysis of cancer exosomes isolated from various types of CM or serums has revealed the existence of surface‐anchored MMPs, glycosidases, and soluble MMPs on the surface or the inside of these vesicles (Das et al., 2019). Future studies should seek to identify other potential cargos of PCa exosomes that are involved in the degradation of bone ECM.

In this study, we used osteoblastic (MDA PCa 2b), osteoclastic (PC3), and mixed (C4‐2) PCa cells to investigate the effects of their exosomes on bone homeostasis. However, regardless of the cell source, PCa exosomes from the three types of PCa cells all promote osteoclast differentiation. One major reason for this discrepancy is presumably the fact that exosomes derived from PCa cells represent only a part of the mechanism that underlies bone homeostasis. As mentioned earlier, osteoblast‐stimulating cytokines (e.g., BMP4 from MDA PCa 2b) and osteoclast‐stimulating factors (e.g., sRANKL from PC3) secreted from PCa cells play an important role in the process. We attempted to investigate this discrepancy by assessing miRNA expression in the PCa cells and the characteristics of exosomes derived from the cells. We found that osteoclastic miR‐92a‐1‐5p and osteoblastic miR‐375 and miR‐148a‐3p were universally expressed in PCa cell lines, which indicate the roles of PCa cells in the bone microenvironment. Furthermore, obvious differences in relative osteogenic index of the three PCa cells may partly explain the differential roles of the PCa cells on bone homeostasis. We also compared the exosome yield and in vivo exosome distribution between groups and observed no differences. Subsequently, we cocultured PCa exosomes with Raw264.7 cells and MC3T3‐E1 cells for a short time to determine differences in internalization. Interestingly, PC3 exosomes were more easily uptaken by Raw264.7 cells and C4‐2 exosomes by MC3T3‐E1 cells, indicating that different PCa exosomes target and function in different target cells despite being all osteoclastic. Further studies on these differences in target cells in vivo and the osteolytic ability of the different PCa exosomes are needed.

Two mechanisms of extracellular vesicle biogenesis have been suggested (Minciacchi et al., 2015; Choi et al., 2015). Exosomes, which represent one nanoscale subpopulation of extracellular vesicles, originate from the endosome system and are released through the exocytosis of multivesicular bodies (MVBs). Other extracellular vesicles are derived from plasma membrane budding and are enriched in plasma membrane proteins. Extracellular vesicle heterogeneity is defined by the biogenesis process, size variation, and cargos of the vesicles (Zijlstra & Di Vizio, 2018). Alix and Tsg101, which are involved in the formation of intraluminal vesicles, are regarded as exosome markers (Bissig & Gruenberg, 2014; Boyiadzis & Whiteside, 2017; Cocucci & Meldolesi, 2015). However, selective markers for extracellular vesicles derived from plasma membrane budding remain to be identified. GW4869, a neutral sphingomyelinase inhibitor that we used in this study, is the most widely used inhibitor of exosome generation; GW4869 acts by blocking the ceramide‐mediated inward budding of MVBs and the release of mature exosomes from MVBs (Essandoh et al., 2015). We selected the term “exosomes” for the extracellular vesicles that we examined in this study based on their morphology, size, marker‐protein expression, and coherence of context.

Collectively, our findings suggest that osteogenic tumor‐derived exosomes promote osteoclast differentiation and inhibit osteoblastogenesis by transferring miRNAs and thus directly degrade the bone ECM. PCa exosomes and the transferred miRNA, miR‐92a‐1‐5p, are critical mediators that disrupt bone homeostasis, degrade the bone matrix, induce pathological bone remodeling, and, ultimately, create an osteoclastic premetastatic niche for tumor growth.

## CONFLICT OF INTEREST

No potential conflict of interest was reported by the authors.

## AUTHOR CONTRIBUTIONS

Lijuan Yu designed and performed most of the experiments and wrote the manuscript; Bingdong Sui designed part of the experiments and revised the manuscript; Weixiao Fan and Lin Lei performed part of the in vitro experiments; Yue Zhang performed part of the in vivo experiments; Lei Zhou, Liu Yang, Yanjun Diao, Zhuo Li, and Jiayun Liu provided technical and resource assistance throughout the project; Xiaoke Hao coordinated the study, designed the experiments and provided funds.

## Supporting information

Supporting information

Supporting information

Supporting information

Supporting information

Supporting information

Supporting information

Supporting information

Supporting information
