# Supplementary material for: Exosomes derived from osteogenic tumor activate osteoclast differentiation and concurrently inhibit osteogenesis by transferring COL1A1‐targeting miRNA‐92a‐1‐5p
Source: J Extracell Vesicles. 2021 Jan 18;10(3):e12056. doi: 10.1002/jev2.12056 (PMC7812369; doi:10.1002/jev2.12056)
Supplement: Supplementary file 1 — Supporting information [file JEV2-10-e12056-s001.docx]

**Supplemental material**

**Table S1.** Primer sequences used for qPCR.

| **Gene** | **Forward (5**ʹ **-3**ʹ**)** | **Reverse (5**ʹ **-3**ʹ**)** |
| --- | --- | --- |
| Gapdh | GGTGAAGGTCGGTGTGTGAACG | CTCGCTCCTGGAAGATGGTG |
| Alp | CACGGCGTCCATGAGCAGAAC | CAGGCACAGTGGTCAAGGTTGG |
| Runx2 | ACTCTTCTGGAGCCGTTTATG | GTGAATCTGGCCATGTTTGTG |
| Ocn | GCTCGGCTTTGGCTGCTCTC | AGCTGCTGTGACATCCATACTTGC |
| Osx | TCGTCTGACTGCCTGCCTAGTG | CTGCGTGGATGCCTGCCTTG |
| Trap | CACTCCCACCCTGAGATTTGT | CATCGTCTGCACGGTTCTG |
| Rank | AGCCTCCGAGCAGAACTGACTC | CTGCCTGTGTAGCCATCTGTTGAG |
| Ctsk | GGCCAGTGTGGTTCCTGTTGG | CCGCCTCCACAGCCATAATTCTC |
| RANKL | CGGGGTGACCTTATGAGAAACTG | CCAGGGAATTTACAAAGTGCACC |

**Figure S1.** Distribution of PCa exosomes *in vivo*. (a) Representative *ex vivo* images at 4, 24, and 48 h. No significant difference in fluorescent pigment distribution among groups was found. (b) No pathological changes were observed in the heart, liver, spleen, kidney, stomach, or intestine at 24 h post-injection. Scale bar = 500 μm.

**Figure S2.** C4-2 and PC3 exosomes facilitate osteoclast differentiation *in vitro*. (a-b) Comparison of exosome yield before and after GW4869 treatment using BCA Protein Assay (a) and NTA (b). Significant decrease in protein content and particle number is shown. (c) Different concentrations of RANKL promoted osteoclast differentiation at distinct levels. Scale bar = 100 μm. (d) Trap staining of BMMs after a 6-day treatment with high or low concentration of exosomes (50 or 25 μg/mL, respectively) in the presence of 30 ng/mL M-CSF and 50 ng/mL RANKL. More Trap^+^ osteoclasts were observed in C4-2 Exos group and PC3 Exos group, compared to Control group. Scale bar = 100 μm. (e) Trap staining of Raw264.7 cells after culturing for 6 days with 100 ng/mL RANKL and 50 μg/mL PCa exosomes. Number of Trap^+^ osteoclasts in C4-2 Exos group and PC3 Exos group was higher compared with Control group. Scale bar = 100 μm. (f) Western blotting of PCa cell lysates and PCa exosomes. RANKL was lowly expressed in PCa cells and no expression was observed in PCa exosomes. (g) mRNA expression of RANKL in PCa cells using qPCR. Low expression of RANKL was observed. Data were analyzed with *t* test and one-way ANOVA with multiple comparison test. *, *P* < 0.05; **, *P* < 0.01; ***, *P* < 0.001.

**Figure S3.** C4-2 and PC3 exosomes inhibit osteoblastogenesis *in vitro*. (a) ALP activity after a 7-day treatment with 40 μg/mL PCa exosomes. Reduction on ALP activity is shown. (b) ALP staining after a 7-day treatment with 40 μg/mL PCa exosomes in osteogenic induction medium. Downregulation was observed in PCa exosomes groups. Scale bars = 5 μm (upper) and 100 μm (lower). Data were analyzed with one-way ANOVA with multiple comparison test. *, *P* < 0.05; **, *P* < 0.01; ***, *P* < 0.001.

**Figure S4.** PCa exosomes target bone *in vivo*. After 4 weeks of exosome injection, (a) BALB/C mice in different groups showed no morphological differences. (b) No change in bone morphology was observed in the mice. (c) X-ray scanning revealed no differences between the groups. (d) Representative H&E-stained sections after 4 weeks of exosome injection. Trabecular separation and decreased trabecular bone area are shown. Scale bar = 150 μm. (e) No pathological changes were observed in the intestine, kidney, liver, lung, or spleen. Data were analyzed with t test. Scale bar = 100 μm.

**Figure S5.** C4-2 and PC3 exosomes induce osteolysis *in vivo*. After 4 weeks of injection of PBS (Control) or C4-2 or PC3 exosomes (10 μg, thrice weekly), micro-CT analysis was performed to determine BMD (a), BV/TV (b), BS/BV (c), Tb.Th (d), Tb.N (e), and Tb.Sp (f) in the harvested tibia. (g) Representative 3D-reconstruction images from micro-CT illustrating trabecular separation. (h-j) Trap-staining images and quantitative results after 4 weeks of injection. Trap^+^ cells were significantly upregulated in C4-2 Exos and PC3 Exos groups. Data were analyzed with one-way ANOVA with multiple comparison test. *, *P* < 0.05; **, *P* < 0.01; ***, *P* < 0.001.

**Figure S6.** Exosomes from PCa-patient serum promote osteoclast differentiation *in vitro*. (a) Comparison of exosome yield by BCA method. More exosomes were isolated in PCa with bone metastasis group. (b) Trap staining of BMMs after 6 days culture with exosomes derived from PCa patient serum. Exosomes both from localized PCa and bone metastatic PCa promoted osteoclast differentiation. Bone metastatic PCa exosomes had a stronger ability on osteoclast differentiation compared with localized PCa exosomes. Six patient samples (three localized PCa and three bone metastatic PCa) were performed in three independent experiments and similar results were obtained. Scale bar = 50 μm. (c) ALP staining of MC3T3-E1 cells after 7 days coculture with Bone Met PCa Exos. Reduction was observed in Bone Met PCa Exos group compared to control. Scale bars = 5 μm (upper) and 100 μm (lower). Data were analyzed using *t* test and one-way ANOVA with multiple comparisons test. *, *P* < 0.05; **, *P* < 0.01; ***, *P* < 0.001.

**Figure S7.** Comparison of PCa cells and PCa exosomes. (a) Differential expression of osteoclastic miR-92a-1-5p in three PCa cell lines. (b-c) Differential expression of osteoblastic miR-375 and miR-148a-3p in three PCa cell lines. (d) Relative osteogenic index of the three cell lines. Relative osteogenic index = relative miRNA expressions of (miR-148a-3p + miR-375 - miR-92a-1-5p). (e) Comparison of the exosome yield of three PCa cell lines. (f) 100 μg Vybrant DID-labeled PCa exosomes were injected to BALB/C nude mice via tail vein. Comparison of fluorescence intensity in bone 24 hours post-injection using *ex vivo* imaging. No significant difference was discovered among groups. (g) Internalization of PCa exosomes in Raw264.7 cells after 90 min incubation. Higher fluorescent intensity is shown in PC3 Exos group. Scale bar = 20 μm. (h) Internalization of PCa exosomes in MC3T3-E1 cells after 180 min incubation. Higher fluorescent intensity is shown in C4-2 Exos group. Scale bar = 20 μm. Data were analyzed using one-way ANOVA with multiple comparisons test. *, *P* < 0.05; **, *P* < 0.01; ***, *P* < 0.001.
