# Supplementary figures and images for: Exosomes derived from osteogenic tumor activate osteoclast differentiation and concurrently inhibit osteogenesis by transferring COL1A1‐targeting miRNA‐92a‐1‐5p
Source: J Extracell Vesicles. 2021 Jan 18;10(3):e12056. doi: 10.1002/jev2.12056 (PMC7812369; doi:10.1002/jev2.12056)

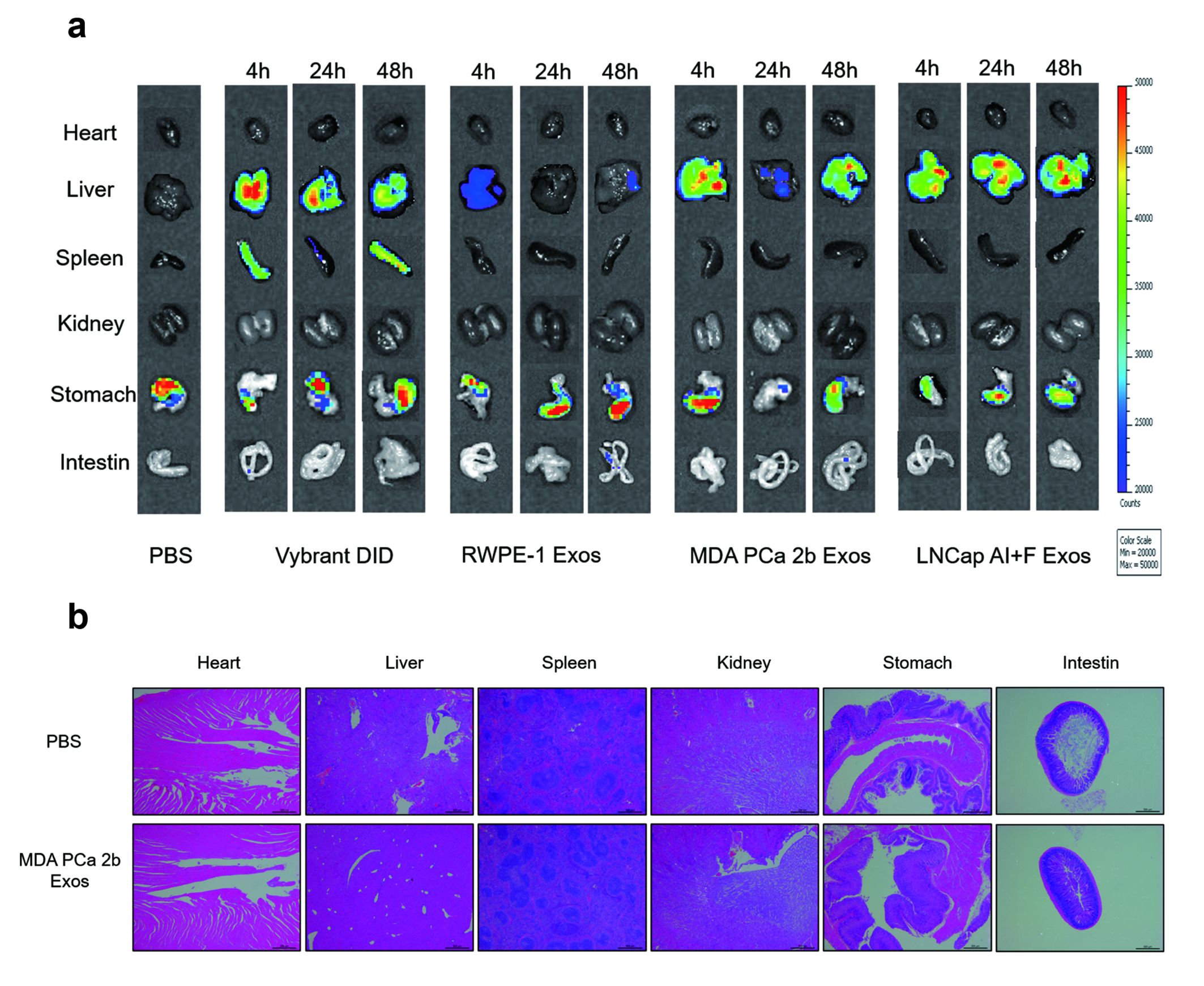

Supplement: Supplementary file 2 — Supporting information [file JEV2-10-e12056-s002.tif]

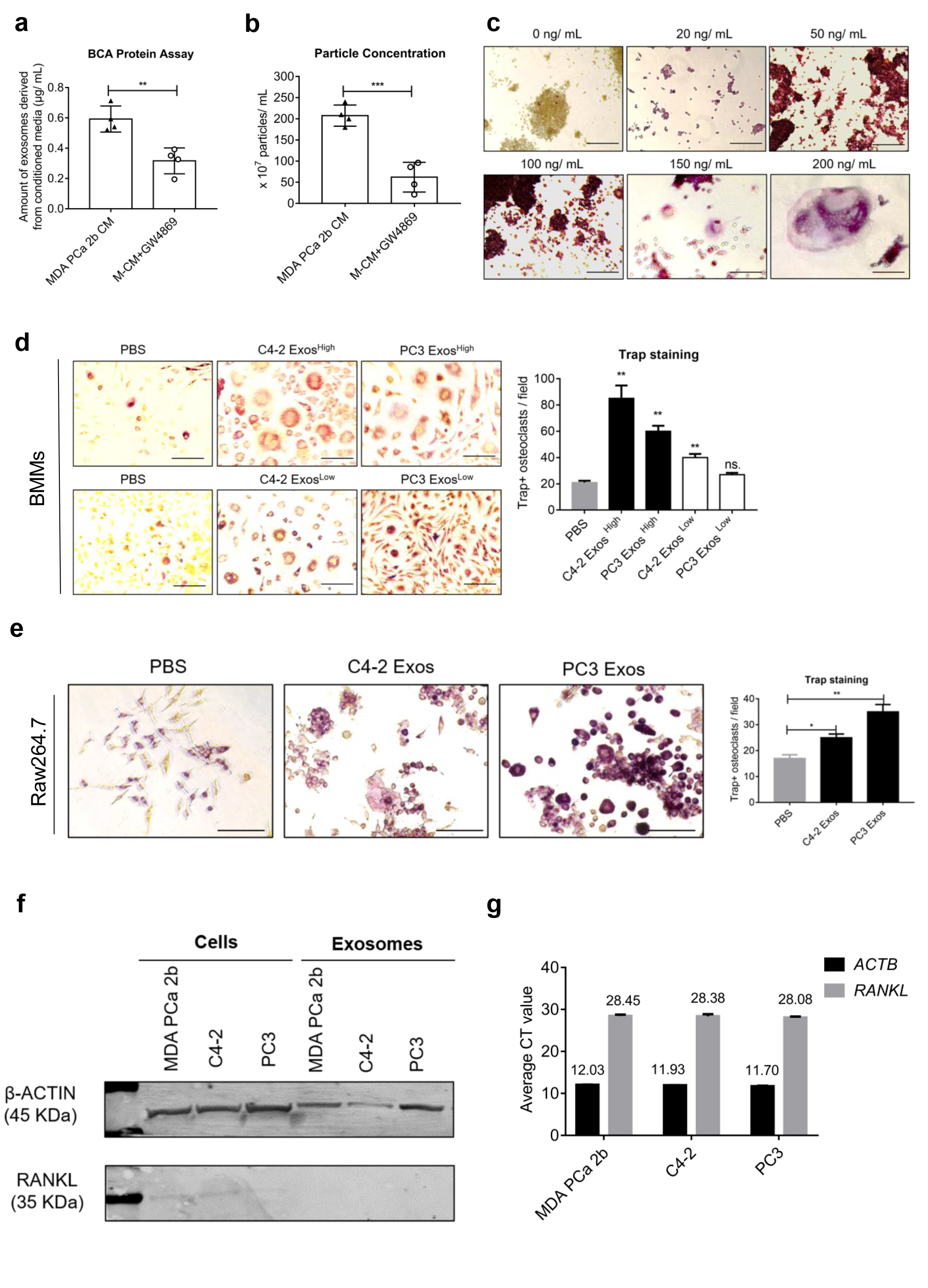

Supplement: Supplementary file 3 — Supporting information [file JEV2-10-e12056-s003.tif]

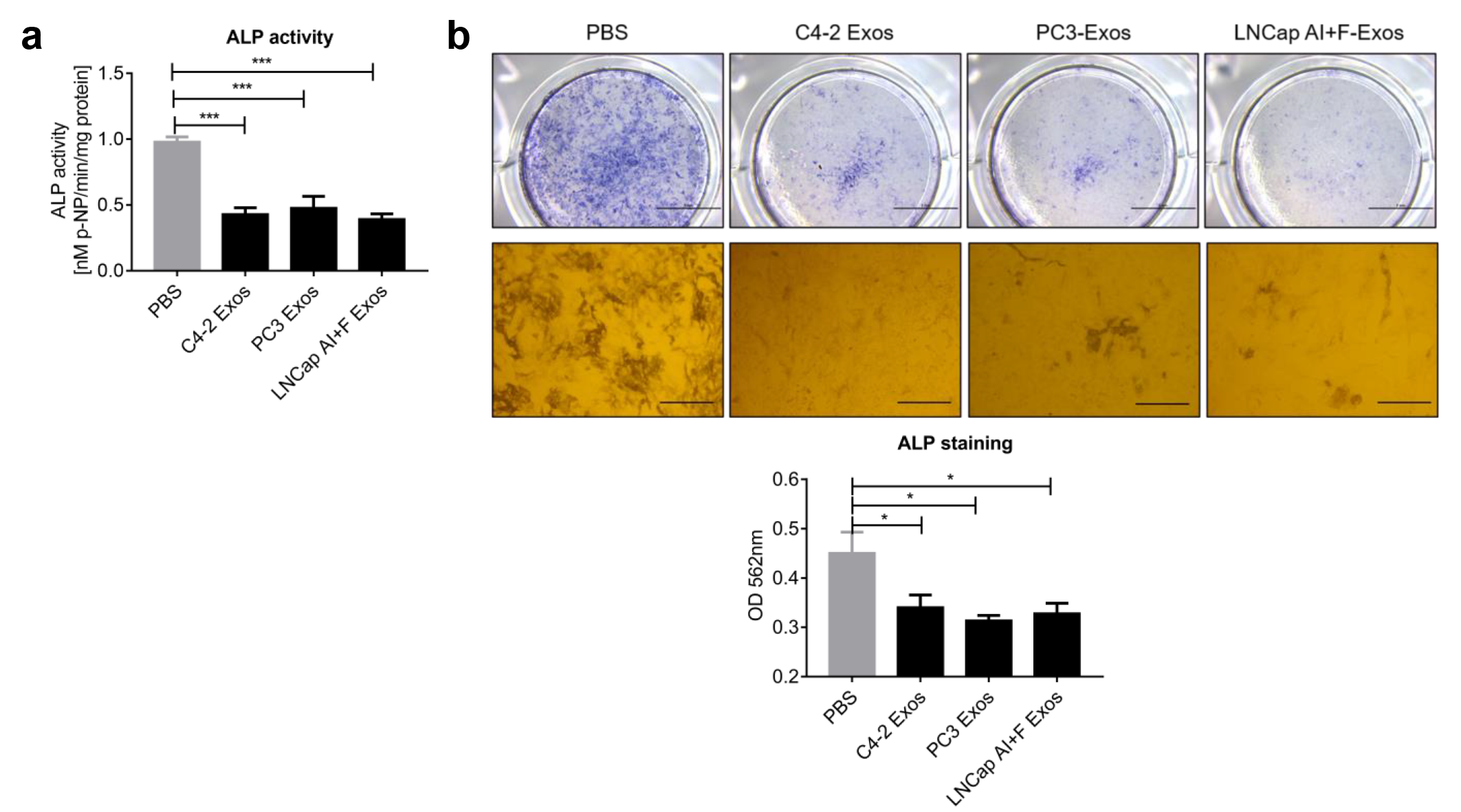

Supplement: Supplementary file 4 — Supporting information [file JEV2-10-e12056-s004.tif]

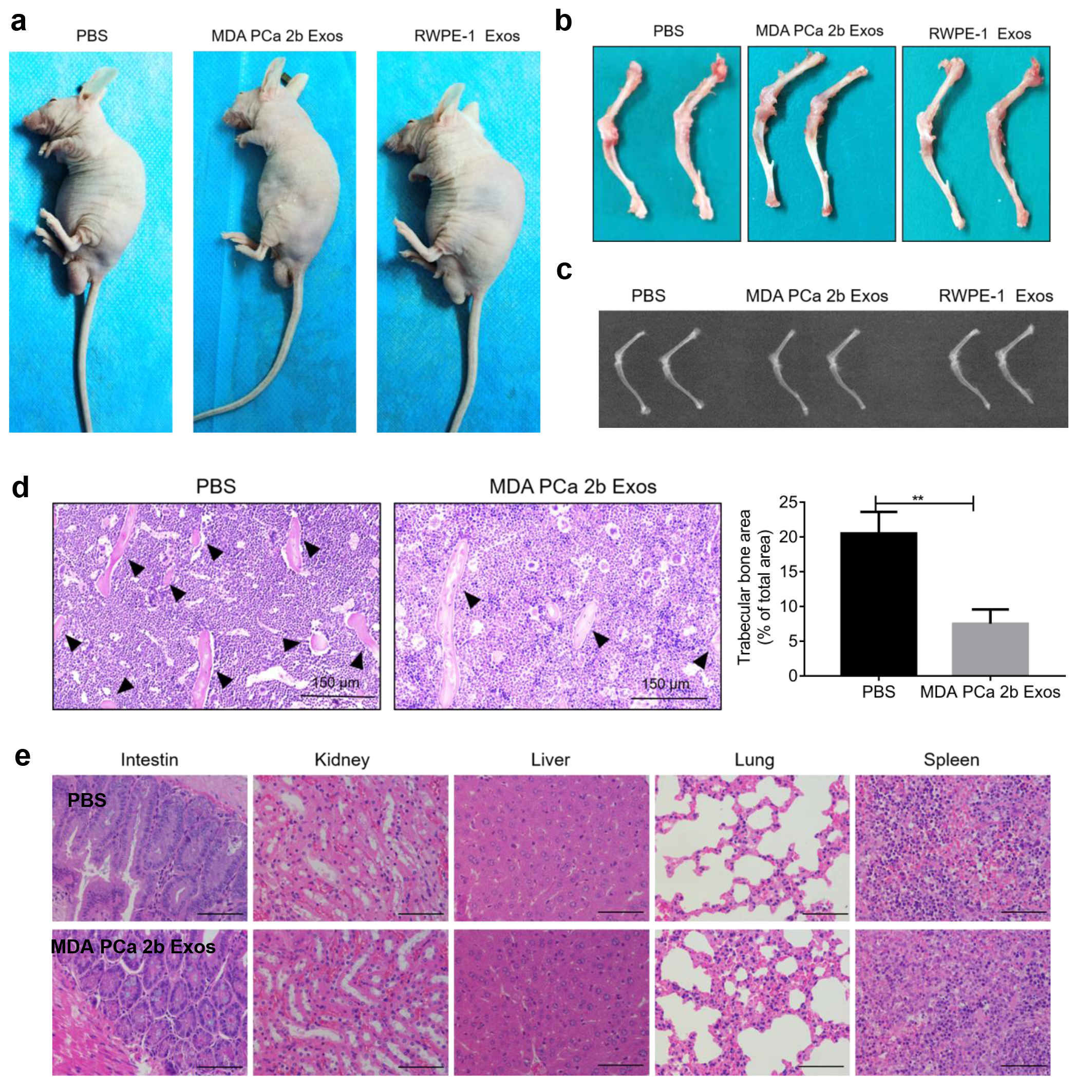

Supplement: Supplementary file 5 — Supporting information [file JEV2-10-e12056-s005.tif]

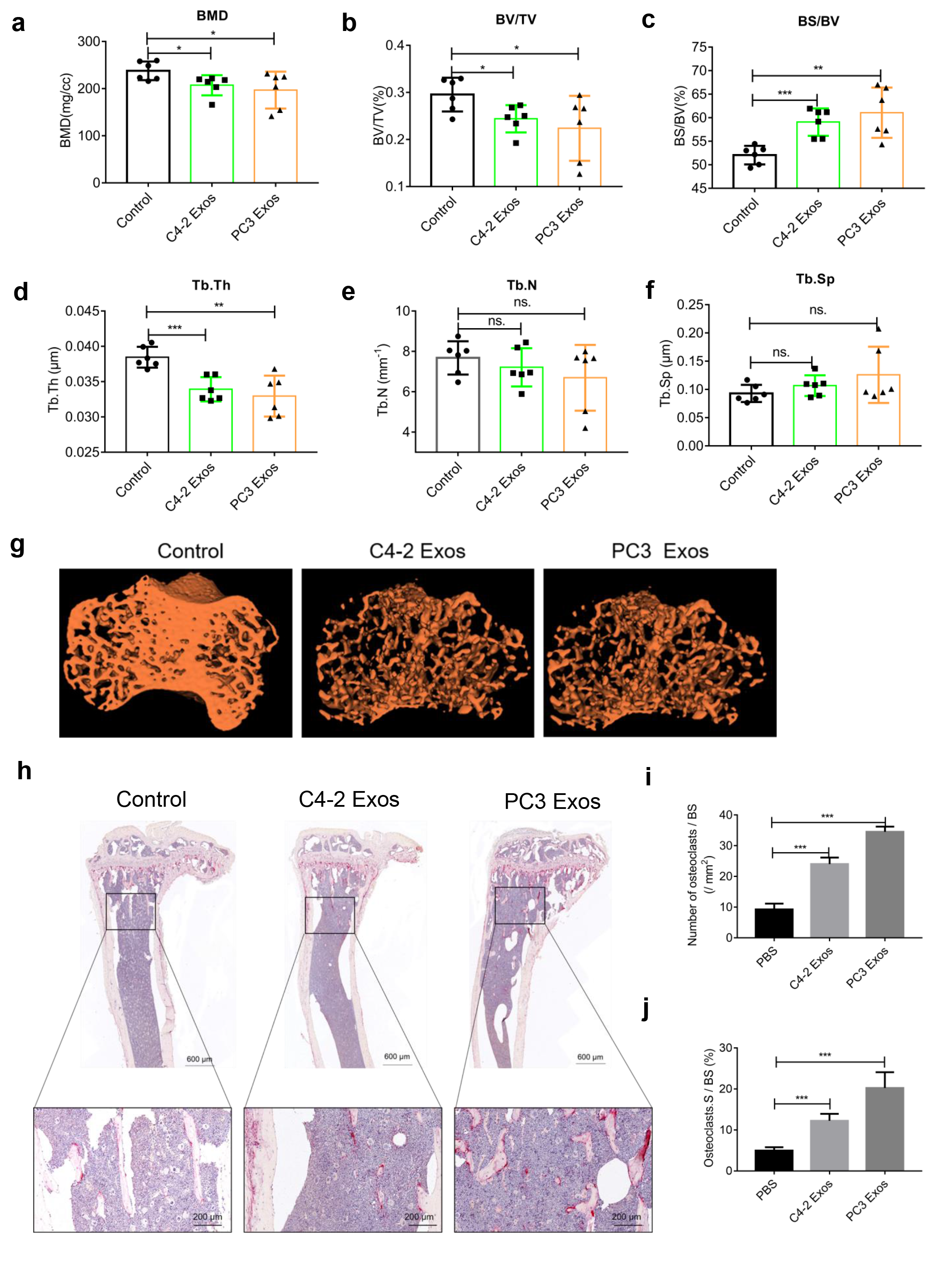

Supplement: Supplementary file 6 — Supporting information [file JEV2-10-e12056-s006.tif]

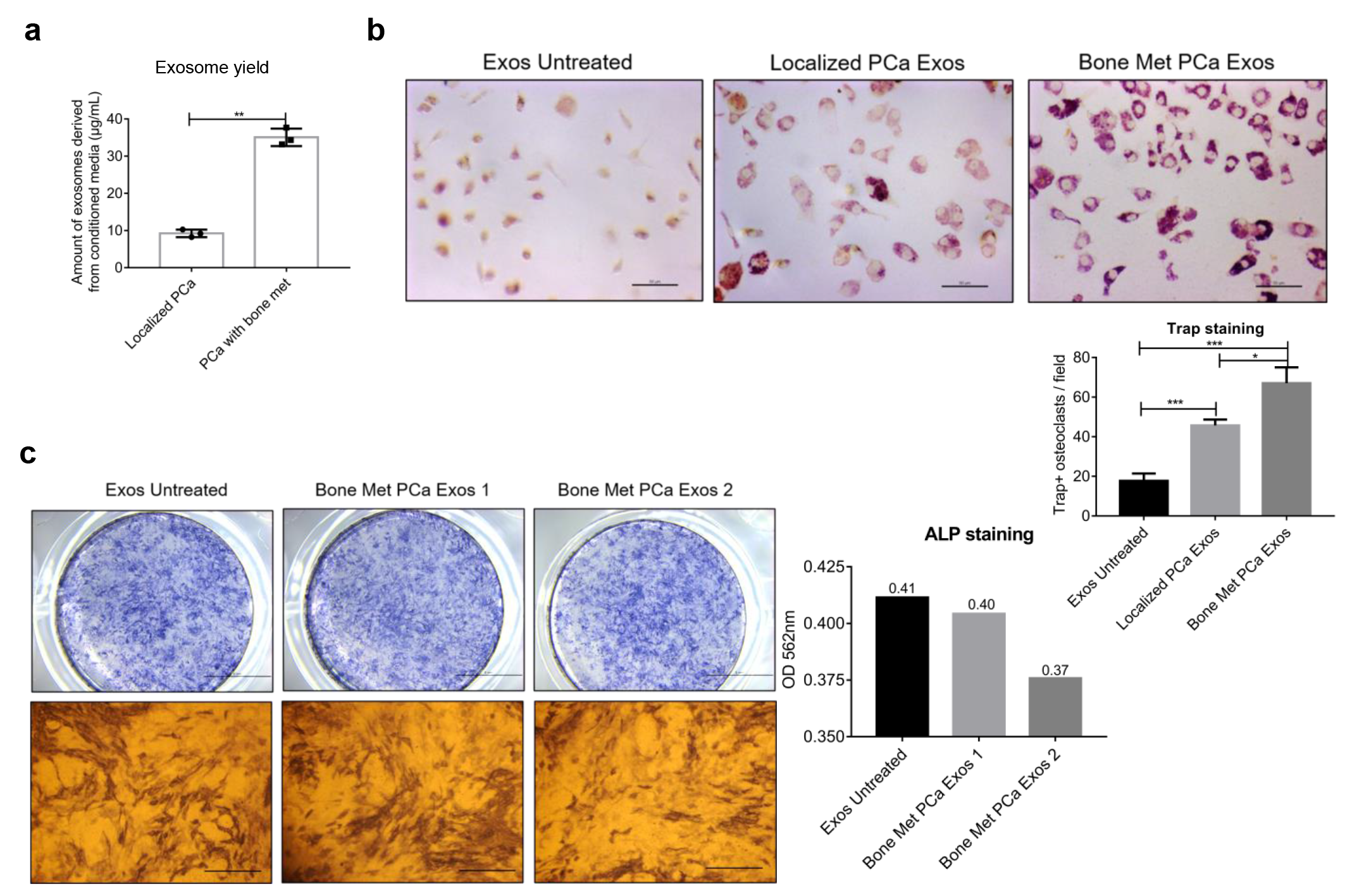

Supplement: Supplementary file 7 — Supporting information [file JEV2-10-e12056-s007.tif]

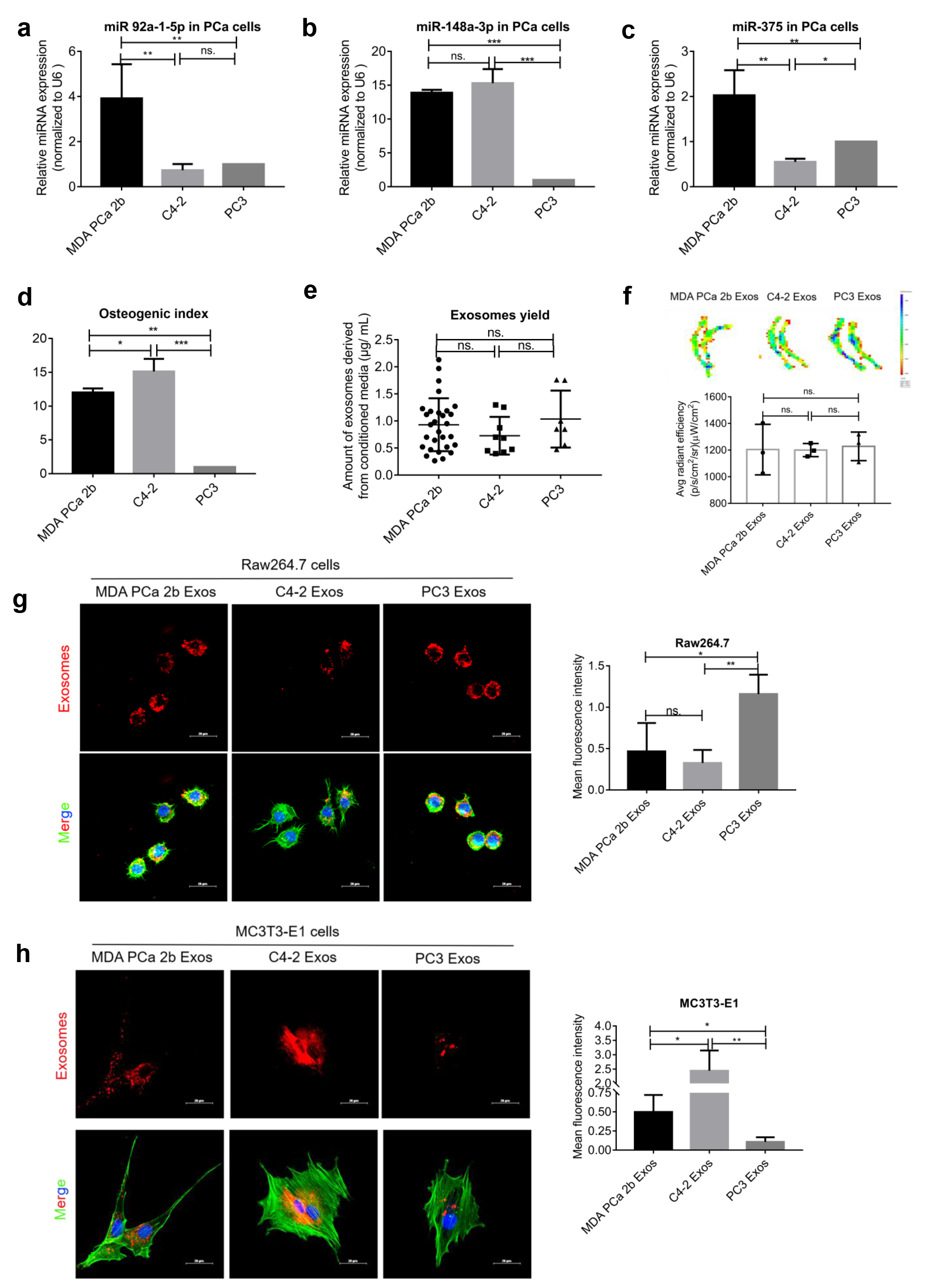

Supplement: Supplementary file 8 — Supporting information [file JEV2-10-e12056-s008.tif]
